# Spatiotemporal Assessment of the TEMPO Formaldehyde Column Retrieval Using the Pandonia Global Network

**DOI:** 10.1029/2025JD044788

**Published:** 2026-01-28

**Authors:** Prajjwal Rawat, Katherine R. Travis, Barron Henderson, James H. Crawford, Laura M. Judd, Mary Angelique G. Demetillo, Tabitha C. Lee, David E. Flittner, James J. Szykman, Lukas C. Valin, Andrew Whitehill, Eric Baumann, Thomas F. Hanisco, Apoorva Pandey, Gonzalo Gonzalez Abad, Caroline R. Nowlan, Xiong Liu, Kelly Chance

**Affiliations:** 1 NASA Langley Research Center, Hampton, VA, USA,; 2 Environmental Protection Agency, RTP, NC, USA,; 3 NASA Goddard Space Flight Center, Greenbelt, MD, USA,; 4 Center for Astrophysics | Harvard & Smithsonian, Cambridge, MA, USA

## Abstract

Launched in April 2023, the Tropospheric Emissions: Monitoring of Pollution (TEMPO), instrument provides for the first time hourly measurements of atmospheric pollutants over most of North America at high spatial resolution (~2 × 4.75 km^2^). This evaluation of TEMPO’s first year demonstrates the capability of total formaldehyde column retrievals (ΩHCHO, version 3) at different locations, seasons, and meteorological conditions. The ΩHCHO product is assessed using 36 ground-based Pandora direct-sun measurements from Pandonia Global Network (PGN) as a reference data set. The 36 PGN sites were chosen for consistency in direct-sun and sky-scan measurement modes. In the first year of operation (Aug 2023–Sep 2024), TEMPO ΩHCHO exhibits moderate to strong agreement at PGN sites in both measurement modes (*R*^2^ = 0.63 to 0.85). TEMPO shows a negligible bias of −2 ± 20% at lower ΩHCHO (<1.0 × 10^16^ molecule cm^−2^) and a larger underestimation of −22 ± 5% at higher ΩHCHO (>1.5 × 10^16^ molecule cm^−2^). TEMPO clearly captures the seasonal variability of ΩHCHO, with summer values being greatest and winter, spring, and fall values being lower by − 62%, − 45%, and − 29%, respectively. TEMPO shows no consistent bias at any time of day with excellent agreement with Pandora for different meteorological conditions. For all hourly differences between TEMPO and Pandora, 96% fall within 1 × 10^16^ molecules cm^−2^. TEMPO provides almost 50% more days with at least one observation compared to observations taken only at 1 p.m., from typical polar-orbiting satellites. These findings confirm the high quality of TEMPO’s ΩHCHO measurements under a wide variety of conditions and show great promise for future scientific applications.

## Plain Language Summary

North America now has the TEMPO geostationary satellite instrument, providing hourly, high-spatial-resolution air quality observations. Geostationary satellites are revolutionizing air quality monitoring by offering continuous hourly data on atmospheric pollutants, which enable in-depth studies of diurnal variations, emission sources, and pollutant transport. Satellite-derived air quality products require careful validation with high quality, ground-based data sets. The TEMPO formaldehyde (HCHO) product demonstrates strong agreement with these data sets and shows significant potential for future scientific applications.

## 1. Introduction

Formaldehyde (HCHO) is an abundant compound with a short lifetime of only a few hours and is sustained by the continued oxidation of volatile organic compounds (VOCs). These precursor VOCs, as well as a small amount of primary HCHO, are directly emitted by natural and anthropogenic activities (Barkley et al., 2013; Fried et al., 2003, 2020; Lee et al., 1998). During the oxidation of VOCs, ozone and secondary organic aerosols (SOA) are produced in addition to HCHO (Jimenez et al., 2009; Seinfeld & Pandis, 2006). Both ozone and SOA (as part of PM_2.5_) are criteria air pollutants that directly affect air quality and public health (NRC, 2004; Silva et al., 2016). HCHO itself is a known carcinogen defined as a hazardous air pollutant (HAP) by the EPA (Zhu, Jacob, et al., 2017). Despite HCHO’s importance, routine ground measurements are sparse and models representing the state of knowledge have difficulty predicting HCHO (Harkey et al., 2021; Skipper et al., 2024). As a result, satellite instruments have long played a large role in our understanding of HCHO.

Space-based monitoring of column HCHO (ΩHCHO) has been possible using ultraviolet radiances for over 30 years beginning with the Global Ozone Monitoring Experiment (GOME) (Chance et al., 1991, 2000). ΩHCHO has also been retrieved from a variety of other instruments including the SCanning Imaging Absorption spectroMeter for Atmospheric CHartographY (SCIAMACHY) (Dufour et al., 2009), GOME-2 (Hewson et al., 2013), the Ozone Monitoring Instrument (OMI) (Ayazpour et al., 2025; De Smedt et al., 2021; Gonzalez Abad et al., 2015), the TROPOspheric Monitoring Instrument (TROPOMI) (De Smedt et al., 2018; Vigouroux et al., 2020), and the Ozone Mapping and Profiler Suite (OMPS) (Nowlan et al., 2023). All of these instruments were mounted on sun-synchronous, polar-orbiting satellites, which typically provide one overpass per day at mid-latitudes.

Satellite observations of ΩHCHO have been used for estimating emissions of non-methane VOCs (NMVOCs) and understanding their impact on ozone production (Barkley et al., 2013; Feng et al., 2024; Millet et al., 2006, 2008; Palmer et al., 2003; Surl et al., 2018; Zhu et al., 2014) and organic aerosols (Liao et al., 2019). Satellite information has provided valuable information on HCHO cancer risk in the US (Zhu, Jacob, et al., 2017). ΩHCHO, combined with nitrogen dioxide (NO_2_) column data, have demonstrated some utility to characterize ozone photochemical regimes (Duncan et al., 2010; Martin et al., 2004), however more recent results demonstrate the measurement complexities and associated limitations on this metric (Schroeder et al., 2017; Souri et al., 2023). The column ratio of glyoxal (CHOCHO) to ΩHCHO has been posed to assess the relative importance of anthropogenic versus biogenic VOC emissions with limited success (DiGangi et al., 2012; Kaiser et al., 2015; Miller et al., 2016). The long-term record of ΩHCHO has also allowed for studies of trends around the world that may indicate changing VOC emissions from a variety of source types (Bauwens et al., 2022; Zhu, Mickley, et al., 2017). These studies were limited to a single observation time per day due to the polar orbiting nature of the instruments.

Geostationary satellites provide daytime hourly observations and are a major advancement in space-based monitoring of air quality. To date, three geostationary instruments have been launched, completing the Geostationary Air Quality (Geo-AQ) constellation over the Northern Hemisphere (CEOS, 2019). The Geostationary Environment Monitoring Spectrometer (GEMS) was launched in 2020 by South Korea and covers much of Asia at ~3.5 × 8 km^2^ resolution for most trace gas and aerosol products (Kim et al., 2020). Sentinel-4, developed by the European Space Agency (ESA), was launched on 01 July 2025 and will provide ~8 × 8 km^2^ resolution observations over Europe (Holmlund et al., 2021). The topic of this assessment is the Tropospheric Emissions: Monitoring of Pollution (TEMPO) instrument, which was launched in April 2023 and covers North America at 2 × 4.75 km^2^ resolution at the center of the Field of Regard (Chance et al., 2019; Gonzalez Abad et al., 2025; Zoogman et al., 2017). TEMPO provides high-resolution spatiotemporal column data on nitrogen dioxide (NO_2_), formaldehyde (HCHO), aerosols, sulfur dioxide (SO_2_), and ozone (O_3_) (Zoogman et al., 2017). TEMPO’s primary goal is to enhance understanding of key elements of tropospheric chemistry, air quality, transport pathways, and their impacts on health and the environment.

The design requirements of the TEMPO instrument, combined with its operational set up (i.e., integration time of ~100 ms for a single observation and number of observations co-added), results in excellent performance at similar spatial resolution to TROPOMI (3.5 × 5.5 km^2^), despite TEMPO’s distant orbit of ~36,000 km (more than 40 times higher than typical polar sun-synchronous orbit). The added benefit of geostationary observations over polar orbiting observations is multiple views per day, enabling better tracking of air pollution changes, thus allowing for timely assessments of air quality outcomes and better identification of pollution sources (Follette-Cook et al., 2015; He et al., 2025; Judd et al., 2018, 2020; Kim et al., 2020; Zoogman et al., 2014, 2017). TROPOMI achieves ΩHCHO precision as low as 0.5 −0.8 × 10^15^ molecule cm^−2^, surpassing its 1.2 × 10^16^ molecule cm^−2^ pre-launch target (Vigouroux et al., 2020). TEMPO targets a precision of 1 × 10^16^ molecule cm^−2^ with 12 co-added observations (Szykman et al., 2023).

Since the selection of the TEMPO mission, the Pandonia Global Network (PGN-https://www.pandonia-global-network.org) expanded the number of Pandora spectrometers across the TEMPO Field of Regard to serve as the primary source of correlative data (Szykman et al., 2019, 2023; Zoogman et al., 2017). The Pandonia Global Network (PGN) provides real-time, calibrated, spectrally resolved UV-VIS raw radiances (Level 0) through a federated network of ground-based Pandora spectrometers, originally developed to support the validation of space-based sensors (Herman et al., 2009). Analysis against Pandora serves as the metric to bring the TEMPO ΩHCHO retrieval to full validation status and readiness for scientific applications (Szykman et al., 2023). This work assesses the quality of TEMPO version 3 ΩHCHO retrievals using carefully assessed Pandora observations during the first year of TEMPO operation. The quality of TEMPO ΩHCHO observations is assessed with respect to location, season, time-of-day, and meteorological conditions.

## 2. Data and Methods

### 2.1. TEMPO Instrument and ΩHCHO Retrieval

The TEMPO instrument, launched aboard the IntelSat 40e satellite on 7 April 2023, represents a significant advancement in the capability to monitor air quality across North America from geostationary orbit. TEMPO has hourly temporal coverage and records spectra between 293 – 494 nm and 538 – 741 nm at ~0.57 nm spectral resolution (Gonzalez Abad et al., 2024). Details regarding the retrieval of TEMPO ΩHCHO are specified in the TEMPO Algorithm Theoretical Basis Document (ATBD, Gonzalez Abad et al., 2025) and briefly summarized below. ΩHCHO retrievals from TEMPO are based on solar backscatter spectra in the 328.5–356.5 nm wavelength range. The retrieval process involves direct spectral fitting of measured radiances to reference radiances to derive differential slant column densities (SCDs), which are subsequently converted to vertical column densities (VCDs) using air mass factors (AMFs). AMFs are derived from a pre-computed look-up table developed for TEMPO using a radiative transfer model that incorporates viewing and solar geometry, surface reflectance, cloud properties, and the Goddard Earth Observing System Composition Forecast (GEOS-CF) a priori profiles (Keller et al., 2021). A background correction is then applied using GEOS-CF-based background columns estimated across TEMPO’s field of regard.

**Figure 1.**
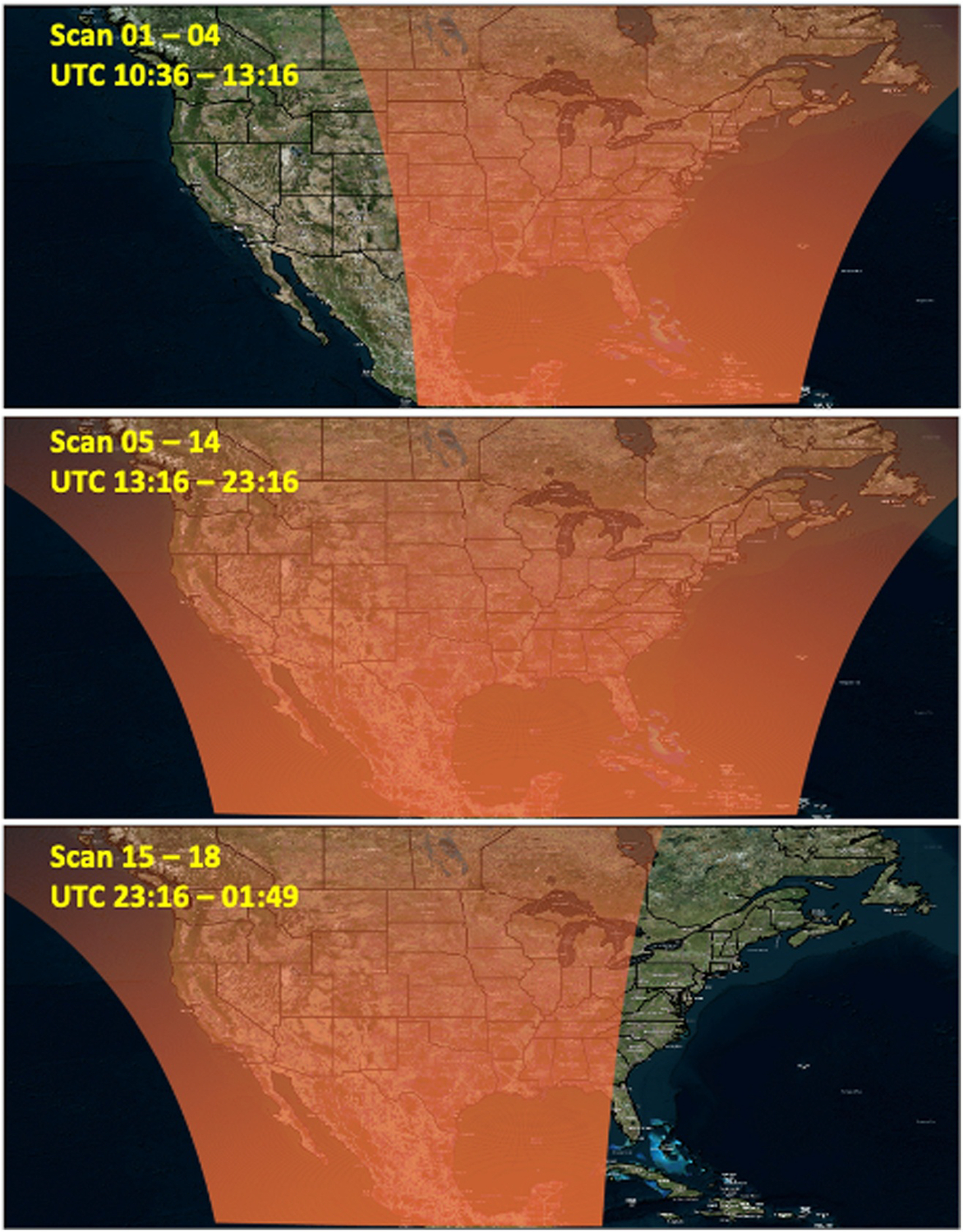
TEMPO scanning coverage over North America on 8 July 2024. The shaded red areas indicate the scanning coverage over the sunlit portion of the sampling domain with annotated scan number and UTC hours.

The scanning pattern of the TEMPO instrument, positioned in geostationary orbit (91°W), is key for understanding diurnal features of pollution sources and their transport mechanisms (Chance et al., 2019). Figure 1 shows TEMPO’s scanning pattern over North America on 8 July 2024. With its detector oriented north–south, TEMPO scans east to west in successive mirror steps, completing full coverage of North America every hour. During the morning and evening hours, shorter 40 min scans are conducted over a more limited east or west region, covering only the sunlit portions of the sampling domain.

This work uses ΩHCHO version 3, level 2 products during the first year of TEMPO operation, between August 2023 and September 2024. This 14-month period is chosen due to data gaps in the first 2 months due to commissioning activities and no data available for 8–22 August 2024. TEMPO data are filtered using the main data quality flag equal to 0 (removing low confidence measurements), solar zenith angle less than 70°, and effective cloud fraction less than 0.2 as recommended by the TEMPO Formaldehyde ATBD (Gonzalez Abad et al., 2025).

### 2.2. Pandonia Global Network Over North America and Spatiotemporal Collocation

The Pandonia Global Network (PGN) provides ground-based Pandora Level 0 radiances, that are processed using common algorithms to produce quality-assured, standardized Level 2 data products, including column densities of key air quality relevant trace gases such as NO_2_, HCHO, O_3_, and SO_2_, with temporal resolutions ranging from seconds to minutes (Herman et al., 2009; Rawat et al., 2025; Spinei et al., 2018). While more commonly used for total column direct-sun (DS) observations of NO_2_, Pandora has recently been able to add HCHO retrievals to their standard processing. Pandora also operates in sky-scan (SS) mode to retrieve profile information and lower tropospheric columns (Cede et al., 2025; Herman et al., 2019). Pandora DS ΩHCHO observations, using the U340 filter, and SS ΩHCHO observations, using both open and U340 filters, alternate throughout the day. When shifting between modes, the pair of consecutive DS and SS observations is separated by less than 5 min (Rawat et al., 2025). While observing schedules can differ, the 5-min separation between consecutive measurements when shifting modes is not affected. As described below, these contemporaneous observation pairs play an important role in evaluating the performance of individual Pandora instruments.

Pandora DS and SS slant columns are retrieved using DOAS analysis in spectral windows of 322.5–359.2 nm and 328.5–359.0 nm, respectively, with HCHO absorption cross sections from Meller and Moortgat (2000). DS ΩHCHO are then obtained via geometrical AMFs and a static reference spectrum, while SS retrieval of the lower-tropospheric column is based on slant column measurements at 60° and 75° pointing zenith angle using a zenith reference spectrum and an empirical algorithm to derive AMFs from O_2_–O_2_ slant columns (Cede et al., 2025). The DS mode in Pandora minimizes the influence of atmospheric factors such as the vertical distribution of gases and aerosols and surface albedo because of the well-defined optical path length, where these factors introduce uncertainties in satellite and SS retrievals (Cede et al., 2006; Herman et al., 2009). However, DS observations can be biased due to instrumental drifts and measurements selected for the instrument specific synthetic reference spectrum (Cede et al., 2025).

Identifying high-quality observations from Pandora is a laborious process and, recently work has shown that automated quality flags reported in PGN data are often too stringent, eliminating large amounts of useful data from consideration. Through careful comparison of contemporaneous Pandora direct-sun and sky-scan observations, Rawat et al. (2025) has developed a method to reassess measurements efficiently and recover data flagged as poor by PGN but having low uncertainty and behavior comparable to data flagged as high-quality for direct-sun/sky-scan comparisons. This filtering recovers Pandora data previously discarded due to strict limits on atmospheric variability and normalized rms of fitting residuals (Rawat et al., 2025). This filtering method increases the available ΩHCHO data for DS and SS for the analysis below by 26% – 90% and 16% – 90%, respectively, over the data flagged with the PGN standard high quality flag. Ongoing routine reports comparing TEMPO to TROPOMI and Pandora for both NO_2_ and HCHO using this filtering methodology are provided for individual sites by the Environmental Protection Agency (ASDC, 2025; Henderson, 2025a, 2025b). These reports show that TEMPO ΩHCHO captures the spatial variability seen in TROPOMI, with strong summer agreement but lower values in winter. Compared to TROPOMI, TEMPO aligns more closely with Pandora.

To address instrument specific performance and data quality issues, Pandora sites used in the validation analysis were selected by comparing DS and SS ΩHCHOs, applying the assumption that both modes are expected to be well-correlated with smaller bias at properly calibrated and maintained sites (Rawat et al., 2025). The contemporaneous measurements of DS and SS ΩHCHO within five minutes of each other are used to identify **reliable** Pandora sites based on the following criteria: (a) *R*^2^ ≥ 0.45, (b) mean differences less than 50% between DS and SS ΩHCHO, and (c) complete seasonal coverage (more than 30 days of data are available for each season in DS) from August 2023 to September 2024. The choice of *R*^2^ ≥ 0.45 represents a clear break point in *R*^2^ values with low-confidence sites having substantially lower correlations. This decision also ensured greater geographic coverage (i.e., Mexico City sites) and the valuable pair of instruments co-located at Greenbelt where NASA calibrates these instruments. The large 50% bias takes into account the expectation of some bias between DS and SS due to SS observing angles not observing the upper free troposphere (Rawat et al., 2025).

Unlike NO_2_, spatial inhomogeneity is not an issue for the DS and SS comparison as HCHO does not experience sharp spatial gradients. ΩHCHO is produced mainly from VOC oxidation and is more spatially homogeneous than ΩNO_2_. Prior work found that observed biases between DS and SS ΩHCHO across Pandora sites showed no systematic dependence on solar azimuth (SAZ), pointing azimuth (PAZ), or PAZ–SAZ (Rawat et al., 2025). It has also been documented that DS-SS differences can be caused by offgassing of Delrin parts in older Pandora instruments. No sites in this analysis contains Delrin parts that caused significant interference in previous HCHO retrievals (Spinei et al., 2021).

The site statistics are tabulated in Table S1 of Supporting Information S1. Of the 85 Pandora sites available under the TEMPO field of regard, 72 sites report in both DS and SS modes. Of these DS sites, 36 satisfied the above criteria, 20 sites are either new or have only a few months of data available due to temporal lags, and 16 did not satisfy the selection criteria. Of these 16 sites, ten had low *R*^2^, four had large bias, and two exhibited both (Table S1 in Supporting Information S1).

Under normal operating conditions, Pandora DS measures ΩHCHO nearly continuously and interspersed with SS measurements, while TEMPO measures every 40–60 min. TEMPO quality-controlled pixels (Section 2.1) within a 10 km radius of a Pandora site are used, with Pandora measurements averaged over 10-min intervals and matched with *a* ±10-min window of the TEMPO overpass time to ensure spatiotemporal collocation. This 10-min averaging reduces noise in Pandora and takes into account the fact that at typical windspeeds (~5 m/s), it could take 20 min for air to traverse the pixels in which the Pandora site is located.

## 3. Comparison Between Pandora and TEMPO ΩHCHO During the First Year of Operation

**Figure 2.**
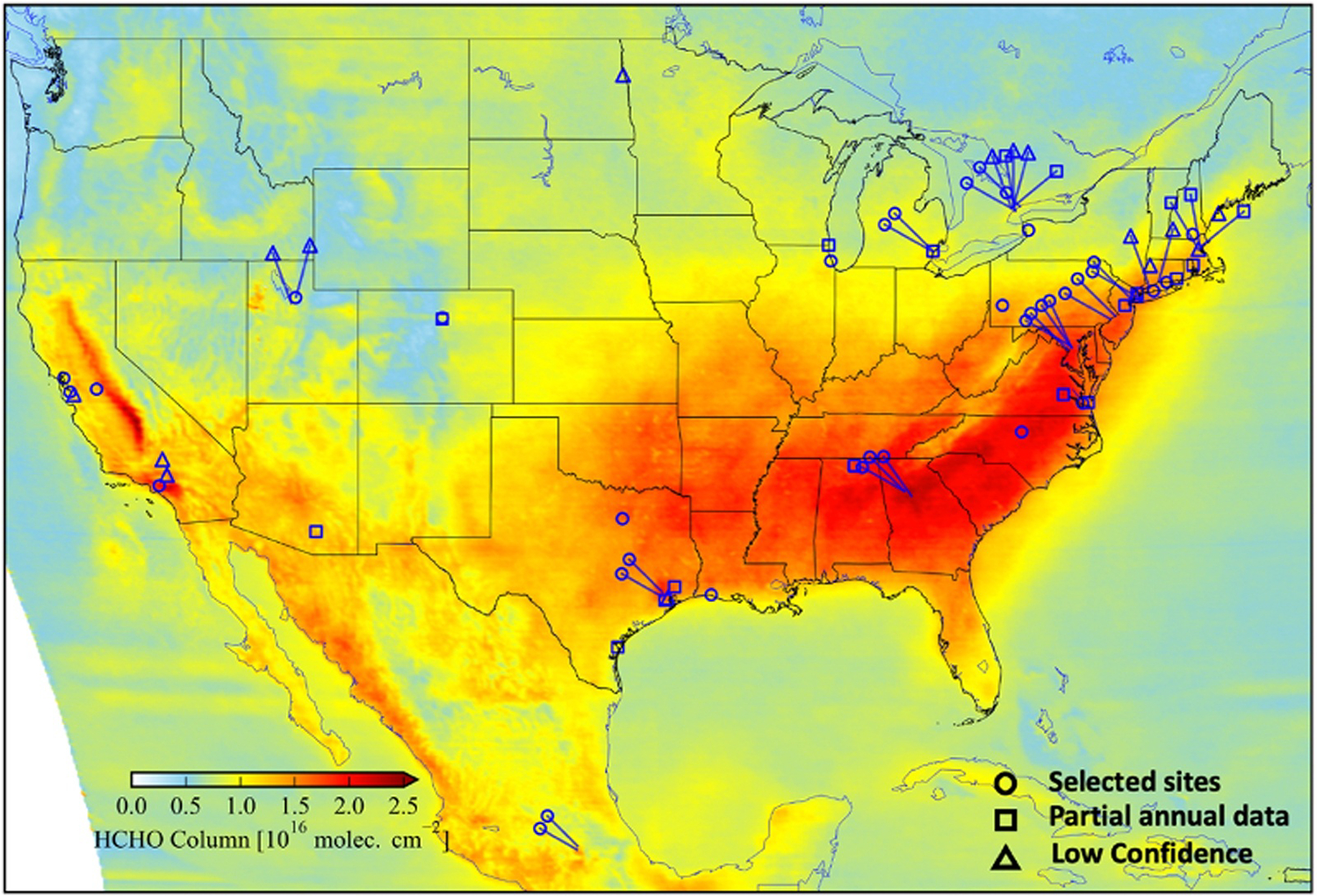
TEMPO v3 average ΩHCHO over North America during July 2024. The circle (36 selected PGN sites), square (PGN sites with partial annual data), and triangle (low confidence PGN sites) designate the locations of Pandora measurements. Pandora sites within a 50 km radius are offset, with a line indicating their actual locations to reduce overlap.

Figure 2 shows a map of the average TEMPO ΩHCHO in July 2024 with the location of the Pandora instruments overlaid. The **circle symbols** represent the 36 selected PGN sites based on the site selection criteria, **squares** indicate the 20 PGN sites with only a few months of data that also meet the site selection criteria, and **triangles** denote the 16 low-confidence PGN sites (Section 2.2). TEMPO shows large ΩHCHO over the eastern US which is consistent with observations from other satellite instruments during the warm months (Abbot et al., 2003; Chance et al., 2000; Palmer et al., 2003; Zhu, Mickley, et al., 2017). Pandora locations are weighted towards regions of greater ΩHCHO (e.g., southeast U.S., coastal northeast, and west coast). Many locations are in or near urban areas, but coverage continues to be expanded into regions needing more representation such as more rural and agricultural locations (SNWG, 2025).

**Figure 3.**
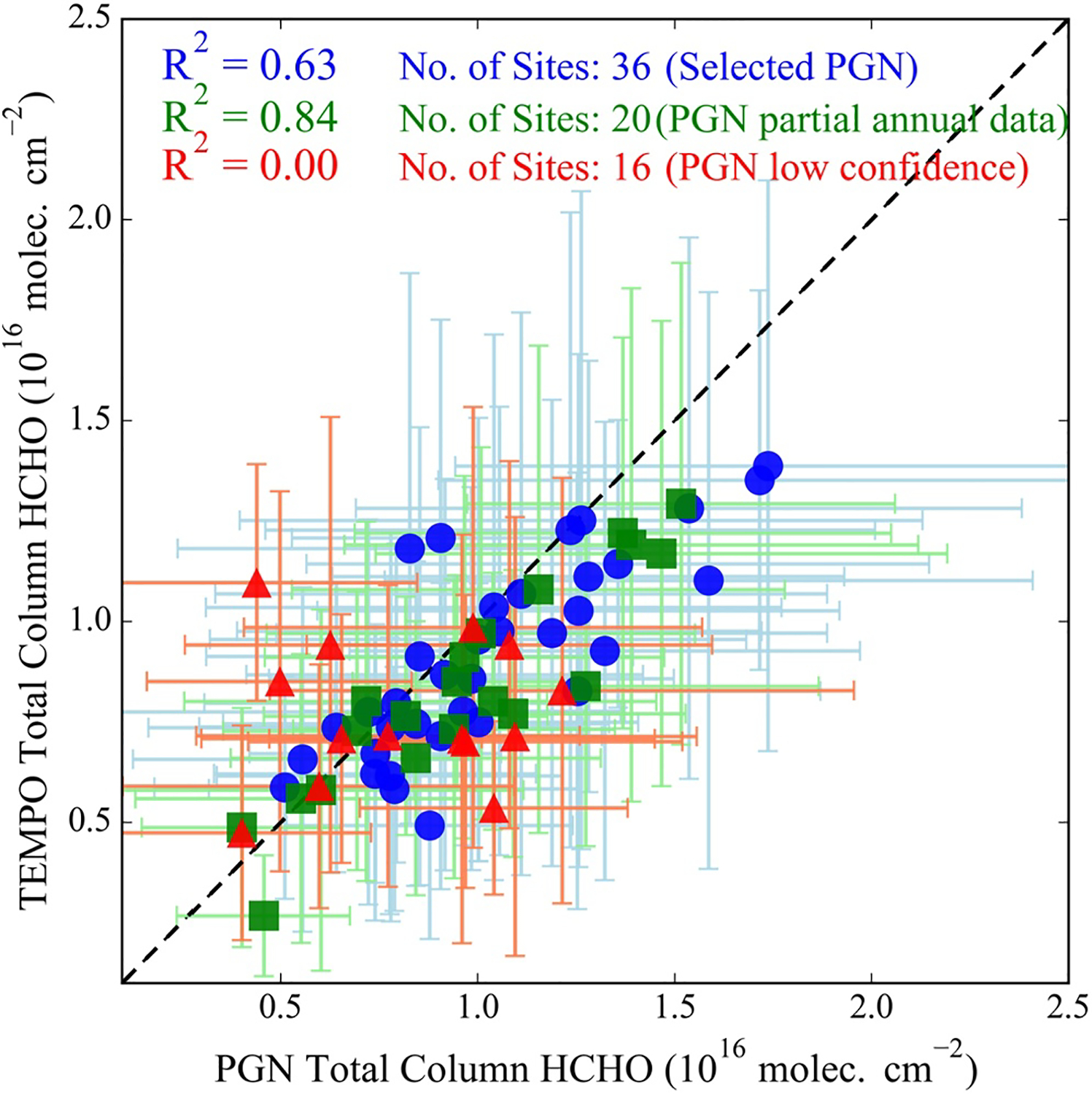
Spatial correlation of TEMPO and Pandora direct-sun ΩHCHO averaged for August 2023 to September 2024 at the 36 selected Pandora sites, 20 sites with partial annual data, and 16 low confidence PGN sites.

Figure 3 shows the site-averaged comparison between Pandora and TEMPO at all sites. Locations are colored by their designated quality status of selected, partial annual data, and low confidence PGN sites. The 36 selected Pandora sites have coefficient of determination (R^2^) or correlation hereafter of 0.63, while PGN sites with partial annual data, mostly limited to summer 2024 measurements, show higher correlations (*R*^2^ = 0.84), mainly due to more favorable DS-SS *R*^2^ and bias values compared to the selected sites (see Table S1 in Supporting Information S1). In contrast, low-confidence sites exhibit poor performance (*R*^2^ of 0.0). Figure S1 in Supporting Information S1 provides the same analysis in Figure 3 but using Pandora SS measurements and shows a strong correlation for the 36 selected sites (*R*^2^ = 0.85), with partial annual data PGN sites showing *R*^2^ of 0.83, and low-confidence sites showing lower correlation (*R*^2^ = 0.49). Pandora SS measurements show a higher correlation for the full year analysis and a similar correlation to DS for the partial annual data sites, but their ΩHCHO values are consistently biased low compared to TEMPO due to their limited vertical extent (below ~3 km). The mean ΩHCHO difference between SS and TEMPO in July 2024 is 2.0 ± 1.7 × 10^15^ molecules cm^−2^, with TEMPO showing higher values. Analysis of TEMPO a priori profiles (GEOS-CF) for the same period indicates that ΩHCHO above 3 km contributes approximately 2.7 ± 0.83 × 10^15^ molecules cm^−2^. Rawat et al. (2025) also reported smaller biases for Pandora DS (−3.8%) and larger biases for SS (up to −30%) relative to airborne GCAS ΩHCHO over Houston. Thus, the observed SS and TEMPO difference can be primarily attributed to the free-tropospheric ΩHCHO contribution. For the subsequent TEMPO ΩHCHO assessments in this work, only the 36 Pandora sites with full-year DS measurements are used.

**Figure 4.**
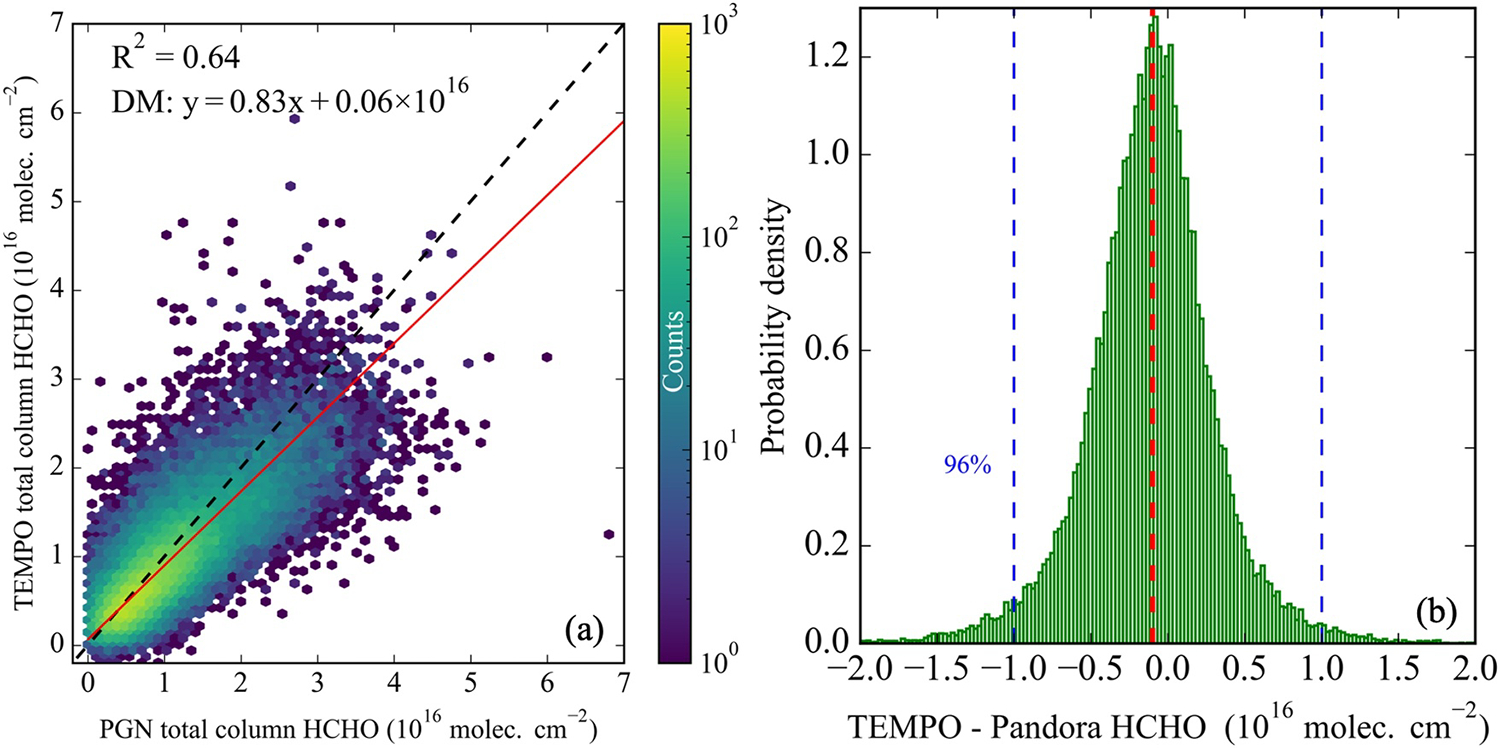
(a) Scatter plot comparing TEMPO with Pandora direct-sun ΩHCHO for August 2023 to September 2024 at the 36 selected Pandora sites for hourly spatio-temporal co-locations. The color scale indicates data density. The intercept and slope were calculated using the Deming regression method, with the assumption of equal uncertainty in both axes. The *x*-axis starts at zero (no negative Pandora column values were observated), while the *y*-axis starts at −0.2 × 10^16^ molec cm^−2^ representing the full range of negative TEMPO observations. (b) Histogram displaying the difference between TEMPO and Pandora ΩHCHO, with TEMPO required precision for 12 co-added observations (1 × 10^16^ molecules cm^−2^) shown by the blue lines and the median bias marked by the red vertical line.

Figure 4a shows all of the data underlying Figure 3 for the 36 selected Pandora sites. Taken as a whole and unaveraged by site, data have an overall spatio-temporal *R*^2^ of 0.64, similar to the site-averaged data in Figure 3. The highest density of points is observed for column amounts below 1.5 × 10^16^ molecule cm^−2^, accounting for 75% of the total collocated observations. The Deming regression indicates that TEMPO underestimates ΩHCHO relative to Pandora DS, with a slope of 0.83. The positive intercept is due to larger underestimation at higher ΩHCHO (see Table S2 in Supporting Information S1). The progression of greater underestimation with increasing HCHO is demonstrated in the following concentration-dependent statistics. At the relatively low concentrations (<1.5 × 10^16^), TEMPO slightly overestimates HCHO by about 3.7%. In the ranges of 1.5–2, 2–2.5, 2.5–3, and >3 × 10^16^ molecules cm^−2^, the underestimation grows to 15.3%, 19.2%, 22.4%, and 29.3%, respectively.

Figure 4b presents the histogram of the differences between TEMPO and Pandora ΩHCHO measurements. The distribution is approximately Gaussian and centered near zero (a median difference of −0.1 × 10^16^ molecules cm^−2^), indicating overall agreement between the two data sets. The majority of data (96%) fall within TEMPO’s required precision (for 12 co-added observations) of 1 × 10^16^ molecules cm^−2^ (Szykman et al., 2023), aligning closely with the 2σ limit of the distribution, while 75% of the differences fall within the 1σ range.

**Figure 5.**
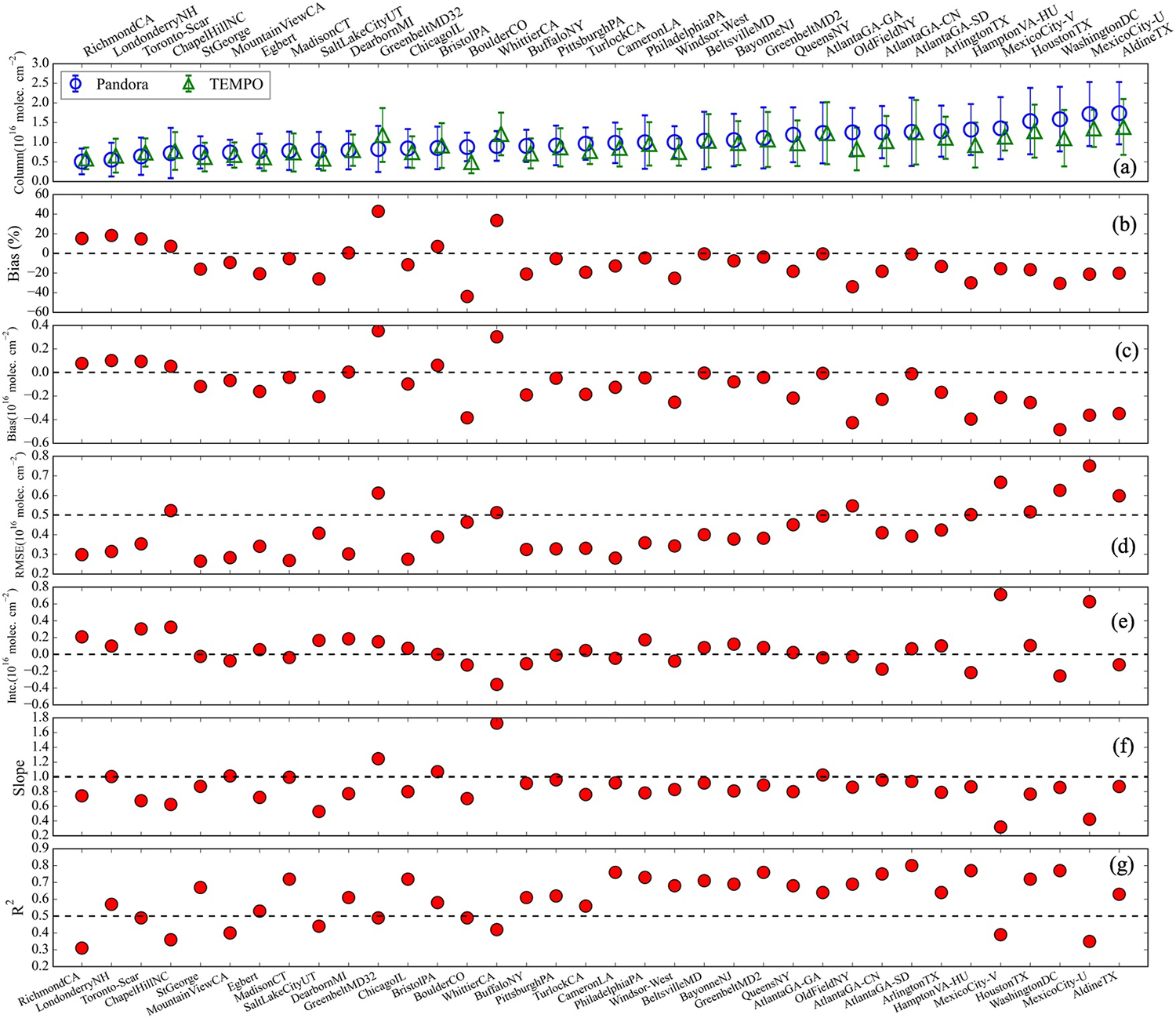
Comparison between Pandora direct-sun and TEMPO v3 in ascending order of Pandora ΩHCHO for (a) average column and standard deviation for August 2023 to September 2024 (b) percentage bias [(TEMPO-Pandora)/Pandora × 100], (c) absolute bias, (c) Root Mean Square Error (RMSE), (d) intercept (Deming regression), (f) slope (Deming regression), and (g) coefficient of determination (*R*^2^). The dashed lines in the subplots indicate the chosen reference for comparing values and are used for explanation in the text.

Figure 5 shows statistics for the 36 selected Pandora sites, arranged in ascending order of average Pandora ΩHCHO. In general, Pandora and TEMPO agree on the relative ΩHCHO magnitude from site to site. Both Pandora and TEMPO show that the highest ΩHCHO is observed in Aldine, TX (Pandora61s1). However, the lowest values differ. Pandora reports the lowest column in Richmond, CA (Pandora52s1), while TEMPO shows the lowest in Boulder, CO (Pandora204s1). The mean percentage bias between TEMPO and Pandora ΩHCHO is within ±30%, except at Greenbelt, MD (Pandora32) and Boulder, CO (Pandora204s1), where TEMPO is +43% higher and −44% lower than Pandora, corresponding to absolute differences of +0.3 × 10^16^ and −0.4 × 10^16^ molecules cm^−2^, respectively (Figure 5c). No relationship is observed between the bias and longitude or latitude of the Pandora sites.

Across all sites, the RMSE for low ΩHCHO is approximately 0.3 × 10^16^ molecules cm^−2^, gradually increasing with higher ΩHCHO values, reaching up to 0.7 × 10^16^ molecules cm^−2^ (Figure 5d and Table S2 in Supporting Information S1). However, the ratio of RMSE to ΩHCHO decreases from ~0.6 at small columns to ~0.3 at larger columns, indicating that TEMPO exhibits better relative performance at higher ΩHCHO despite the larger absolute errors. The *R*^2^ between TEMPO and Pandora at individual sites varies from 0.32 (Richmond, CA) to 0.80 (Atlanta-SD), with the 29 of the 36 sites exhibiting *R*^2^ greater than or equal to 0.5 (Figure 5g).

In general, TEMPO ΩHCHO amounts are in better absolute agreement at lower column values with underestimates at higher column amounts as also shown in Figure 4a. For the 19 sites with average Pandora ΩHCHO < 1.0 × 10^16^ molecules cm^−2^ (TEMPO’s required precision), TEMPO has a minimal negative bias of −0.03 ± 0.17 × 10^16^ molecules cm^−2^ (−2 ± 20%) and RMSE of 0.3 ± 0.1 × 10^16^ molecules cm^−2^. For the 13 sites with average Pandora ΩHCHO between 1.0 and 1.5 × 10^16^ molecules cm^−2^, TEMPO exhibits a negative bias of −0.16 ± 0.14 × 10^16^ molecules cm^−2^ (−13 ± 11%) and an RMSE of 0.4 ± 0.1 × 10^16^ molecules cm^−2^, while for the 4 sites with Pandora ΩHCHO > 1.5 × 10^16^ molecules cm^−2^, TEMPO has a notable negative bias of −0.36 ± 0.1 × 10^16^ molecules cm^−2^ (−22 ± 5%) and an RMSE of 0.62 ± 0.08 × 10^16^ molecules cm^−2^.

The intercept of the relationship between Pandora and TEMPO is below 0.4 × 10^16^ molecules cm^−2^ for most sites. There are 20 sites with slopes between 0.6 and 0.9. The low slopes at higher column values for the two Mexico City Pandora sites indicate that Pandora exhibits greater day-to-day variability than TEMPO in ΩHCHO (Table S2 in Supporting Information S1). The lower *R*^2^ values with TEMPO, along with poor agreement between Pandora DS and SS observations (see Table S1 in Supporting Information S1), also suggest that both Mexico City sites are low-performing.

TEMPO biases are similar to or lower than reported biases in previous satellite validation studies. Vigouroux et al. (2020) assessed TROPOMI ΩHCHO biases relative to ground-based measurements, where TROPOMI overestimated low ΩHCHO (<0.25 × 10^16^ molecules cm^−2^) by +26% and underestimated high ΩHCHO (>0.8 × 10^16^ molecules cm^−2^) by −30%. De Smedt et al. (2021) assessed OMI and GOME-2 ΩHCHO against ground-based observations, showing a low bias of up to 40%. The first geostationary satellite, GEMS, accurately captures spatiotemporal variations across Asia, but consistently underestimates ΩHCHO by −30% to −40% (Fu et al., 2025; Kim et al., 2020; Lee et al., 2024). ΩHCHO from OMPS-NPP and OMPS-N20 also agree well with ground-based data with negative biases (−15% ± 4%) at higher columns and positive biases (20% ± 6%) at cleaner sites (Kwon et al., 2023). OMI Collection 4 ΩHCHO shows good correlation (*r* = 0.83) against ground based FTIR measurements and biases ranging from −8% over polluted sites to +20% over cleaner sites (Ayazpour et al., 2025). While the common underestimation of ΩHCHO at high concentrations across multiple satellites is not understood, individual satellite studies have explored the role of coarse a priori vertical profile shapes, reduced sensitivity due to aerosol and cloud shielding, and satellite footprint size (Boeke et al., 2011; Lorente et al., 2017; Zhu et al., 2016a). Unlike previous studies that used a collocation radius of 20–40 km (De Smedt et al., 2021; Kwon et al., 2023; Vigouroux et al., 2020), this analysis shows that a 10 km radius is sufficient to achieve comparable results for TEMPO.

The analysis in Figure 5 highlights one set of co-located Pandora instruments, the two Pandora instruments in Greenbelt, MD (Pandora #2 and #32), that disagree on the magnitude of Pandora DS ΩHCHO. Table S1 in Supporting Information S1 shows that Pandora #32 has a lower *R*^2^ between DS and SS mode than Pandora #2 (*R*^2^ = 0.49 vs. 0.80). Pandora #2 is in better agreement with TEMPO (bias of −3.6%) compared to Pandora #32 (bias of 43%). The SS measurements from both instruments are highly correlated (*R*^2^ ~ 0.78), while DS measurements exhibit a much lower correlation (*R*^2^ ~ 0.12). This indicates that the discrepancy arises from the DS measurements of #32 rather than differences in viewing geometry or local heterogeneity. These Pandora pairs emphasize the uncertainties that Pandora contributes to the comparison with TEMPO even with careful site selection and the benefit of having Pandora instruments in proximity. Notably, Pandora #32 had known issues with temperature control of the spectrometer and instrument resolution during the comparison period, as confirmed through discussions with the instrument operator. Other notable issues include a 33% overestimate at Whitter, CA. This site has one of the lowest DS-SS biases (11%) which could indicate that DS observations are underestimated at this site. Low correlations at the Mexico City sites are also likely related to Pandora data quality where DS-SS correlations were on the lower end of the selection criteria, and Pandora data shows much greater variability than TEMPO (Figure 5a).

The relationship between Pandora DS versus SS performance compared with the TEMPO versus Pandora DS performance, is evident using all 72 Pandora sites from Table S1 in Supporting Information S1. Figure S2a in Supporting Information S1 shows that the *R*^2^ between TEMPO and Pandora DS improves as the *R*^2^ between Pandora DS and SS increases. Similarly, Figure S2b in Supporting Information S1 shows that the normalized RMSE (NRMSE) between Pandora DS and TEMPO improves with increasing *R*^2^ between Pandora DS and SS observations. Since Pandora SS and DS are expected to be well-correlated with minimal bias at well-calibrated sites, poor validation results may, in some cases, reflect issues with Pandora performance rather than TEMPO. Figure S2c in Supporting Information S1 shows that large biases between TEMPO and Pandora DS typically correspond to large biases between DS and SS, indicating that an issue in one mode drives poor validation results, not heterogeneity resulting from different viewing angles.

## 4. Seasonal Comparisons Between Pandora and TEMPO ΩHCHO

**Figure 6.**
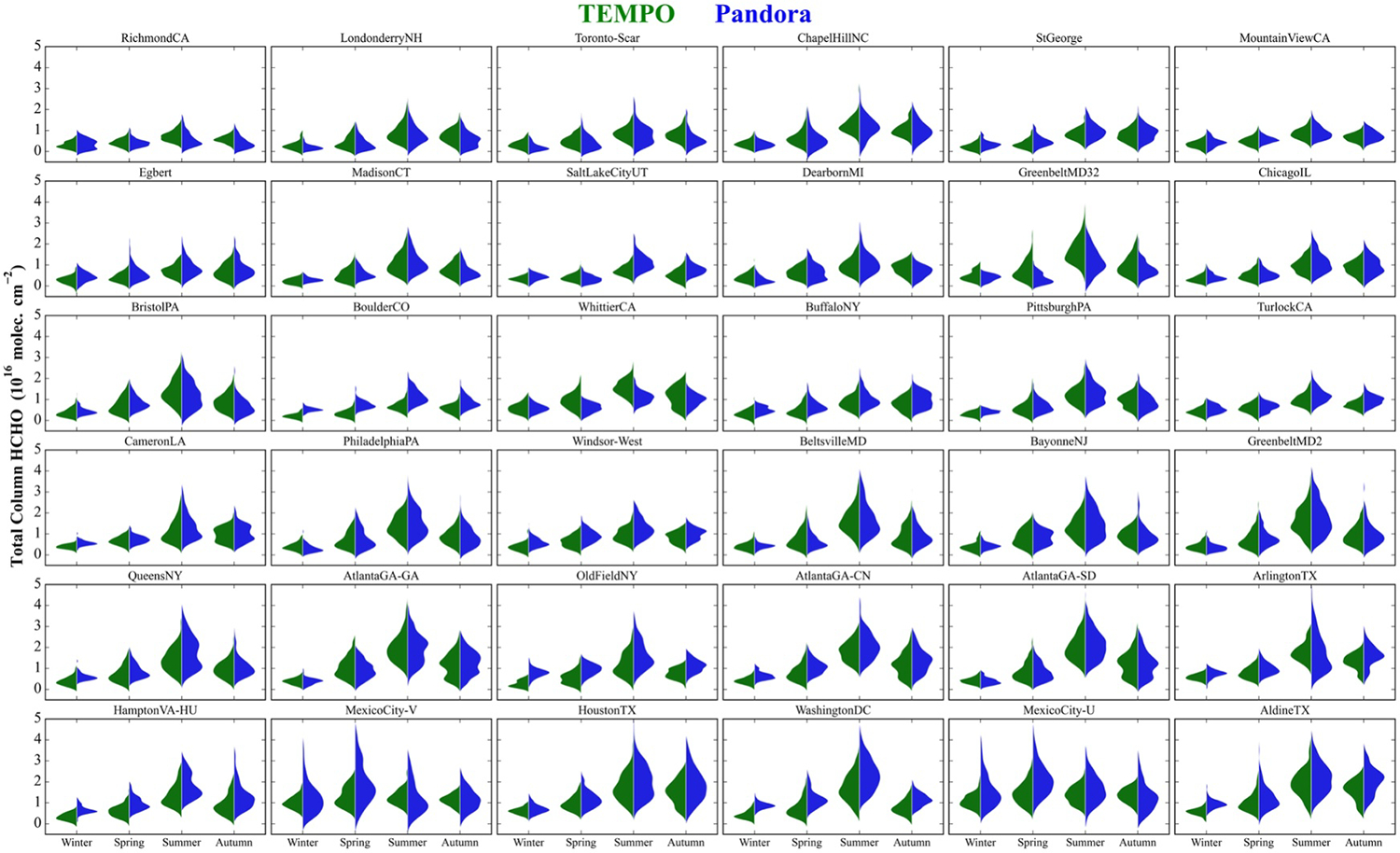
Seasonal variation of ΩHCHO from TEMPO v3 (green) and Pandora direct-sun (blue) measurements during August 2023 to September 2024 for hourly spatio-temporal co-locations. The seasons are described as Winter (DJF), Spring (MAM), Summer (JJA), and Autumn (SON), respectively. The sites are arranged in the same order as shown in Figure 5.

Figure 6 shows distributions of TEMPO and Pandora ΩHCHO in each season in the same site order as Figure 5. Overall, TEMPO seasonality agrees well with Pandora. During the winter season, most of the sites have low average ΩHCHO (<0.5 × 10^16^ molecules cm^−2^). The exceptions are the low latitude Mexico City sites where both TEMPO and Pandora show higher ΩHCHO but with a larger range for Pandora compared to TEMPO. Both Mexico City sites exhibit relatively weak seasonality in ΩHCHO. This is expected due to lower seasonal variations in solar radiation and temperature, along with persistent local emissions. This weaker seasonality has also been observed in FTIR measurements (Rivera et al., 2021).

Pandora and TEMPO show good agreement in ΩHCHO seasonality, with median values in winter, spring, and autumn lower than in summer by −62%, −45%, and −29% for TEMPO, and by −66%, −48%, and −28% for Pandora. During summer, the peak season for photochemical activity and biogenic emissions, all sites show maximum average ΩHCHO for both TEMPO and Pandora (see Figure 6). The exception is Mexico City, which peaks in spring during the dry season.

Figure S3 in Supporting Information S1 shows the percent bias by sites for each season. In summer, 26 sites exhibit biases within ±20%), three sites show larger positive biases, and the remaining seven sites show larger negative biases, in both cases between ±45%. The differences between TEMPO and Pandora remains consistent in sign across seasons for 23 sites, with 5 sites showing a persistent overestimation in TEMPO and 18 sites showing a consistent underestimation. Sites with lower Pandora ΩHCHO (≤0.7 × 10^16^ molecules cm^−2^) tend to show larger positive biases in TEMPO retrievals during autumn and winter, whereas sites with higher ΩHCHO (>1.0 × 10^16^ molecules cm^−2^) exhibit greater negative biases during all seasons. Both Pandora and TEMPO exhibit similar seasonal patterns in ΩHCHO distribution, with a broader spread and higher concentrations during the summer and autumn months (Figure S4 in Supporting Information S1). The overall correlation (*R*^2^) between Pandora and TEMPO ΩHCHO improves during warmer seasons due to the larger ranges of the observed values. Mexico City sites show moderate correlations with consistently similar column ranges across seasons, representative of the low latitude location and consistent availability of sunlight across seasons (Figure S4 in Supporting Information S1).

Figure S5 in Supporting Information S1 shows the differences between TEMPO and Pandora for different seasons as a function of solar zenith angle (SZA). No distinct pattern in the differences is observed with respect to SZA across the seasons, suggesting that the impact of solar geometry on the column measurements does not significantly affect the observed discrepancies. Similarly, when analyzing the seasonal variation along TEMPO AMF (Figure S6 in Supporting Information S1), no major difference between TEMPO and Pandora ΩHCHO is observed, except for a TEMPO underestimation at larger AMF values that is largest in summer months. Variations in profile shapes, aerosol loading, and surface reflectance can significantly impact AMFs and the retrieved ΩHCHO (Kwon et al., 2017; Lorente et al., 2017), underscoring the need for future studies to fully investigate the causes of these biases.

## 5. Diurnal Comparisons Between Pandora and TEMPO ΩHCHO

A major advancement of geostationary observations is the ability to collect data over all daylight hours. This also increases the likelihood of obtaining data on any particular day, when a single overpass may be obscured by cloud. For an eastern Pandora site (BayonneNJ), there are 174 days (between August 2023 and September 2024) of TEMPO observations at 1:00 p.m. local time, which increases to 257 days with at least one TEMPO observation during the study period (total days 427). Loss of observations includes poor retrieval quality and cloudy pixels. The TEMPO cloud filter applied here is strict (cloud fraction <0.2), which may be relaxed in future TEMPO versions as the cloud retrieval improves (Wang et al., 2025).

**Figure 7.**
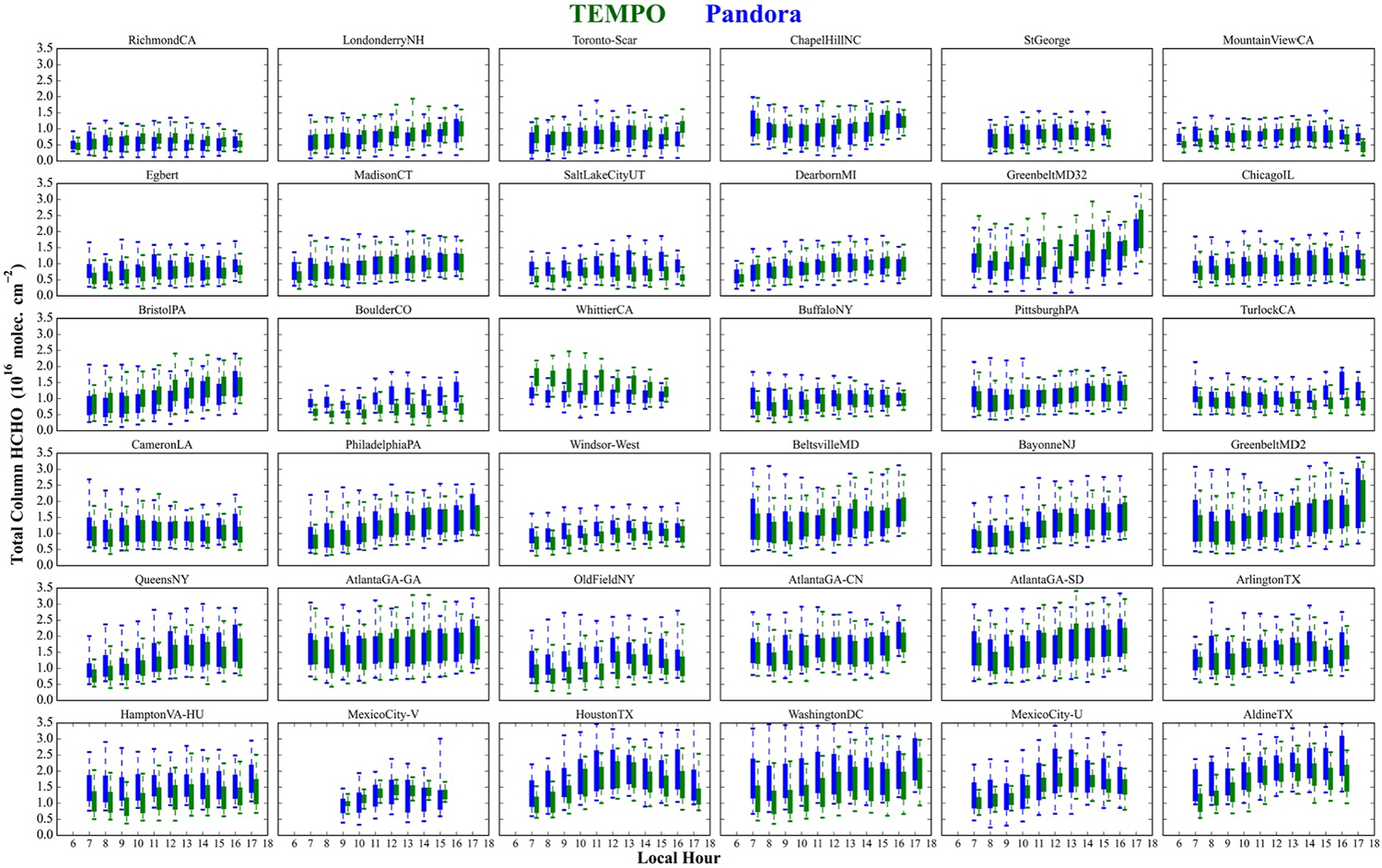
Box-plots of average diurnal variation of ΩHCHO from TEMPO v3 (green) and Pandora direct-sun (blue) measurements during May to October (ozone period) for hourly spatio-temporal co-locations. Only hours with more than 20 matchups are included. The sites are arranged in the same order as shown in Figure 5. The box plot shows the median and the 25th and 75th percentiles, while the whiskers represent the 5th to 95th percentiles.

Figure 7 shows the average diurnal cycles at each selected Pandora site for the defined ozone season of May to October, when HCHO variability is closely linked to ozone formation resulting in poor air quality (Schroeder et al., 2017; Travis et al., 2022). Several distinct patterns in diurnal HCHO profiles are observed across sites, with no bias in TEMPO occurring for any particular diurnal shape. There are apparent biases in the absolute ΩHCHO at some sites, with TEMPO consistently reporting higher values throughout the day over Whittier, CA and Greenbelt, MD (#32). In contrast, Boulder, CO; Old Field, NY; Hampton, VA; and Washington, DC exhibit lower ΩHCHO in TEMPO throughout the day compared to Pandora.

**Figure 8.**
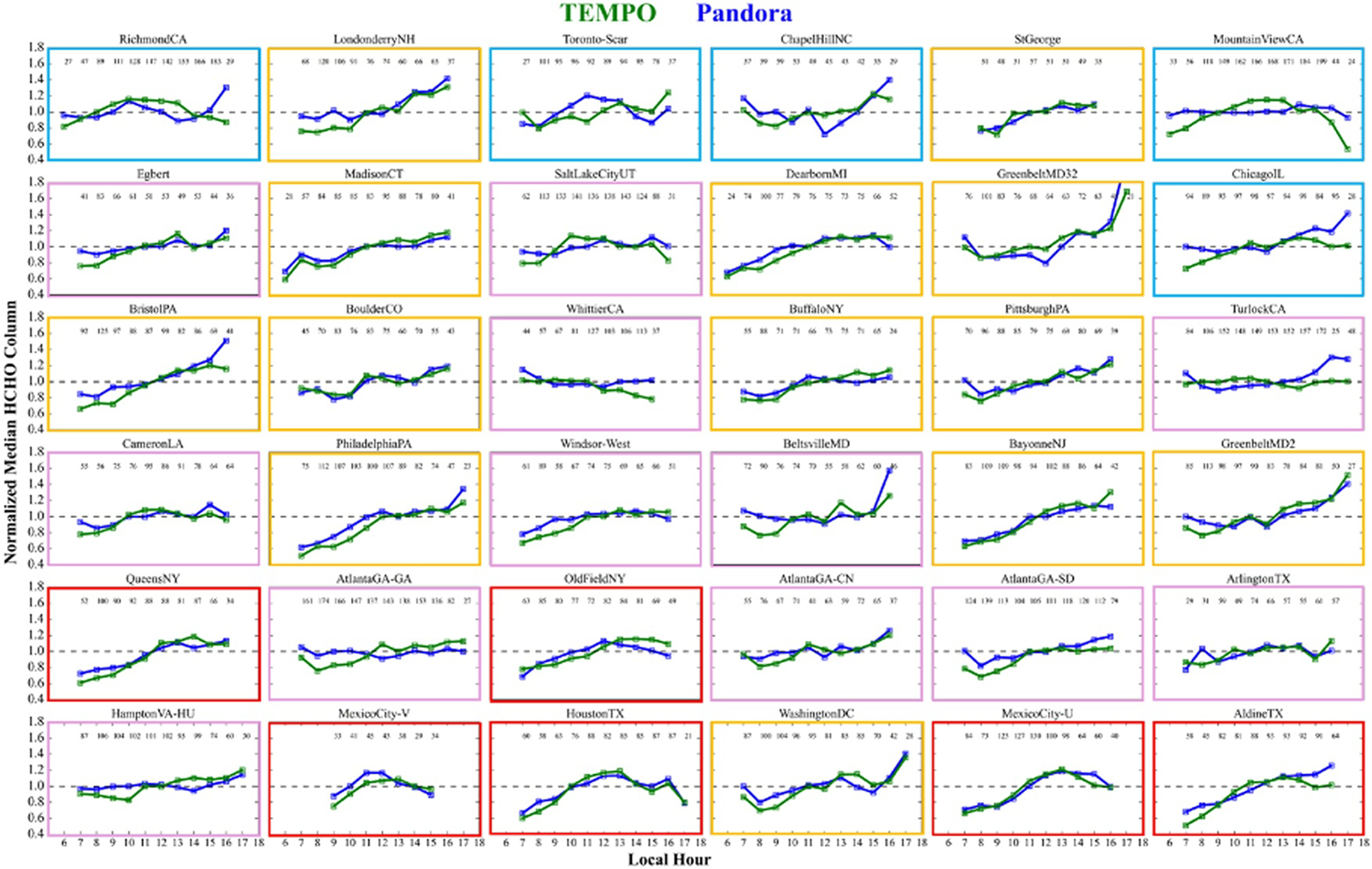
Normalized diurnal variation of ΩHCHO from TEMPO (green) and Pandora direct-sun (blue) measurements during ozone period (May to October) for hourly spatio-temporal co-locations. The subplots are categorized into distinct groups based on their diurnal patterns, with red, yellow, magenta, and cyan borders indicating different characteristic behaviors given in Table 1. The number of matchups at each hour is also annotated in the top of each subplot. The sites are arranged in the same order as shown in Figure 5.

To better analyze the diurnal trends of Pandora and TEMPO, the normalized diurnal trends of ΩHCHO are compared by scaling hourly median values by the median of all hours for each site and shown in Figure 8. Sites are grouped in Figure 8 based on similarities in their diurnal variations as described in Table 1. The classification of normalized diurnal profiles while somewhat subjective was based primarily on their overall shape in Figure 8.

Pandora sites located in Mexico, Texas (Aldine and Houston), and the Northeastern US exhibit HCHO diurnal variability characterized by increasing column amounts peaking in the afternoon (red, *n* = 6), or evening (yellow boxes, *n* = 13). In contrast, sites in the Southeastern and intermountain west (e.g., Atlanta, GA; Atlanta, GA-CN; Atlanta, GA-SD; Hampton, VA; Salt Lake City, UT; Boulder, CO), show little to no significant diurnal variation in ΩHCHO in both Pandora and TEMPO (magenta, *n* = 12), with some differences observed in the morning and evening hours. Additionally, five Pandora sites exhibit marked differences in diurnal behavior between TEMPO and Pandora, mostly for lower HCHO regions. These include two sites in California (Richmond, CA; MountainView, CA), and sites in ChapellHill, NC; Chicago, IL and Toronto-Scarborough. For the California sites, TEMPO captures a midday high i.e. absent in Pandora, whereas other Pandora sites show larger discrepancies in early morning and late evening hours, particularly Chicago, IL.

As diurnal variation is expected to be driven by the lower troposphere, SS observations can also be used to look at the normalized diurnal variation of HCHO between Pandora and TEMPO as shown in Figure S7 of Supporting Information S1 for the 36 selected Pandora sites. Most of the diurnal trends are similar between Figure 8 and Figure S7 in Supporting Information S1, however Pandora SS at ChapellHill, NC and Chicago, IL are more consistent with TEMPO, while over Salt Lake City, UT; Buffalo, NY; AtlantaGA-CN; AtlantaGA-SD; ArlingtonTX; and GreenbeltMD32, exhibit larger discrepancies in SS compared to DS. The inconsistent SS diurnal patterns at AtlantaGA-CN and AtlantaGA-SD largely result from sparse seasonal coverage (e.g., no SS data at Conyers from June to mid-August 2024 and gaps at South DeKalb in Aug–Oct 2023 and June–July 2024), whereas DS data were more consistently available. Salt Lake City, UT; Buffalo, NY, and ArlingtonTX also have limited SS coverage, contributing to diurnal discrepancies. There are consistent discrepancies in the diurnal behavior between both Pandora SS and DS and TEMPO at Mountain View, CA which could be due to an issue with the Pandora site that was not captured by our criteria or a large error in the TEMPO retrieval at this location. The above analysis of TEMPO versus Pandora diurnal variability highlights the value of independent direct-sun and sky-scan observations, not only to enhance confidence in Pandora measurements but also to better diagnose diurnal trends. Over all, there is no consistent evidence for a systematic bias in TEMPO in any part of the day.

## 6. Performance Under Different Meteorological and Aerosol Loading Conditions

Validation across a variety of conditions is key to using ΩHCHO to understand VOC emissions. Variation in ΩHCHO retrievals across a range of temperature conditions provides understanding of the response of VOC emissions to different emissions sources (Zhu et al., 2014). Removing the temperature dependence related to biogenic emissions has allowed for the detection of anthropogenic emission source trends such as increased oil production and agricultural activity (Zhu, Mickley, et al., 2017). Anticorrelation with temperature has been shown to indicate increased use of wood burning in wintertime (Green et al., 2021).

**Figure 9.**
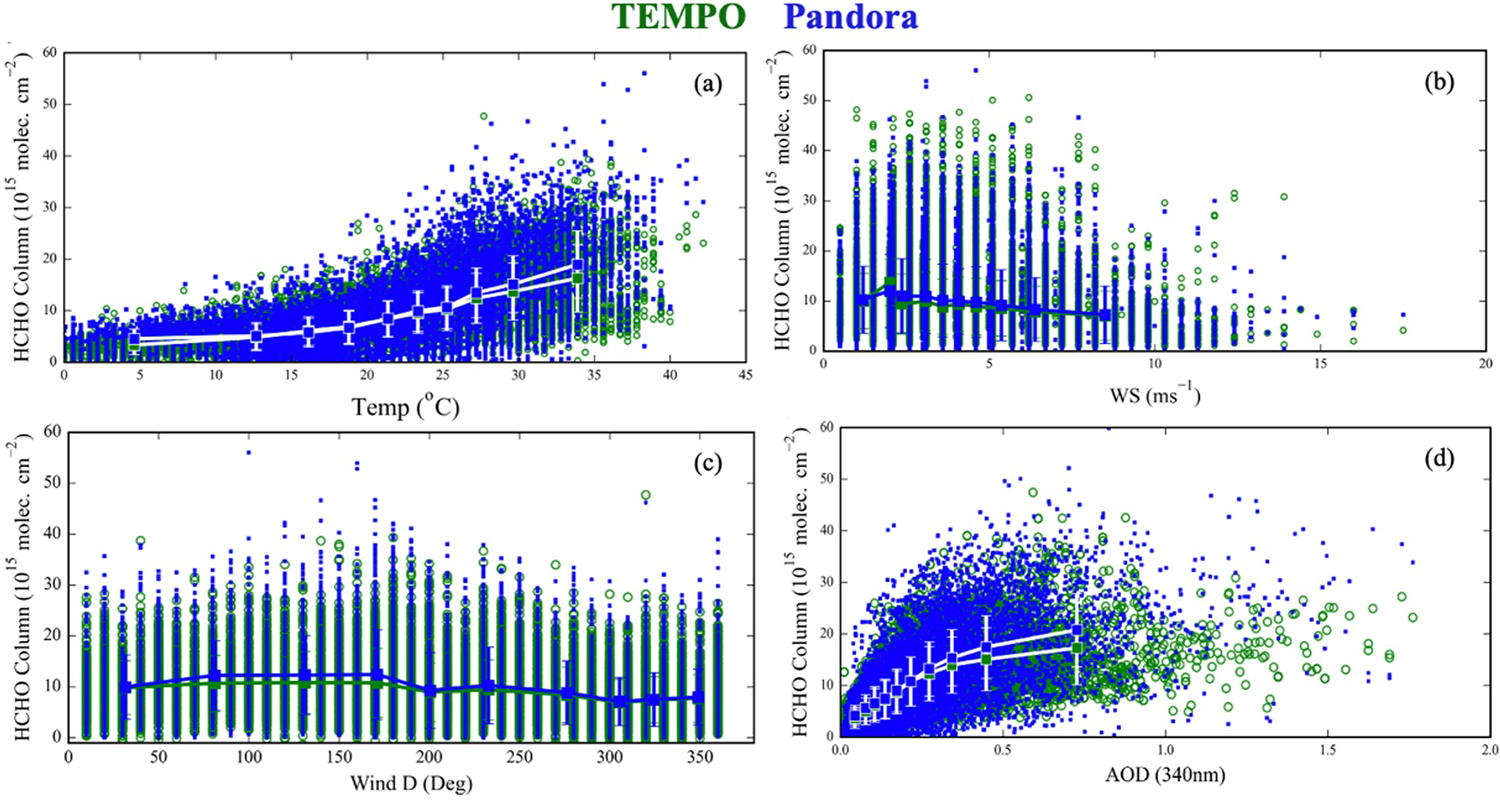
All hourly co-located TEMPO (green) and Pandora direct-sun (blue) ΩHCHO along different meteorological parameters (co-located met obtained from NOAA) and co-located AERONET AOD (a) temperature, (b) wind speed, (c) wind direction, and (d) AOD. The mean is calculated at 10 decile bins of each parameter.

Figure 9a illustrates the distribution of Pandora and TEMPO ΩHCHO across temperature ranges derived from collocated temperature data from the NOAA Integrated Surface Database for August 2023 to September 2024 (NOAA, ISD, 2024). Below 20°C, no significant variation in ΩHCHO is observed, while an increase in abundance occurs at higher temperatures, driven by enhanced biogenic emissions of HCHO precursors, mainly isoprene, and enhanced photochemical activity. The temperature response is consistent between Pandora and TEMPO. There is a minimal departure of <2.7 × 10^15^ molecules cm^−2^ (14%) from agreement at temperatures >25°C. Figure 9b shows the distribution of ΩHCHO across different wind speeds. Larger columns are observed at lower wind speeds due to stagnation, with no biases associated with wind speed, or wind direction (Figure 9c). Lower wind speeds are also associated with higher temperatures (Figure S8 in Supporting Information S1), indicating stronger emissions and potentially clearer sky conditions that favor HCHO production.

Atmospheric aerosols, particularly from fire activities, can significantly affect AMF calculations for satellite-based HCHO retrievals, with impacts reaching up to 30% in regions experiencing heavy smoke (Jung et al., 2019). The TEMPO ΩHCHO retrieval does not explicitly include AOD, but its effects are implicitly accounted for through the use of TEMPO-retrieved cloud properties in the AMF calculations (Gonzalez Abad et al., 2025). Figure 9d shows the distribution of ΩHCHO across different aerosol loading from 17 collocated AERONET sites (within 10 km radius of PGN) (Gupta et al., 2024). Both TEMPO and Pandora observations show increasing ΩHCHO values with rising AOD, with minimal difference (<10%) under AOD below 0.5. At higher AOD levels (>0.9), mostly occurring at the Mexico City, TEMPO ΩHCHO values are lower by 0.6 × 10^16^ molecules cm^−2^ (~26%), compared to Pandora, possibly due to reduced sensitivity to near-surface HCHO caused by aerosol absorption effects (Jung et al., 2019; Lorente et al., 2017).

## 7. Summary and Conclusions

The Tropospheric Emissions: Monitoring of Pollution (TEMPO) sensor, launched in April 2023, represents the first generation of geostationary space-borne UV-VIS spectrometers designed to monitor atmospheric trace gases over North America. This study evaluates the TEMPO ΩHCHO version 3 product between August 2023 and September 2024, focusing on capabilities of TEMPO to capture regional, seasonal, and diurnal variations across its field of regard. Validation of TEMPO ΩHCHO measurements was conducted using data from Pandora spectrometers that are part of the Pandonia Global Network (PGN).

Careful site assessment of the Pandora network for all 85 sites in the TEMPO field of regard was performed, taking advantage of the expectation that the two modes of operation, direct-sun and sky-scan, will be well-correlated and exhibit smaller bias at a properly maintained and calibrated site. This analysis requires the use of a new filtering methodology (Rawat et al., 2025) that increases the available data for this analysis by up to 90% for some sites. Of the 72 sites with both direct-sun and sky-scan observations, there were 36 sites that passed site selection criteria. These sites spanned a wide range of ΩHCHO, with the lowest values observed in the West and less polluted areas (<1 × 10^16^ molecules cm^−2^), to the highest concentrations regions (>1.5 × 10^16^ molecules cm^−2^) in Mexico City, Texas, and Washington, DC. There is high value in Pandora instruments in similar locations to help identify outliers that may be due to poor calibration.

The comparison between TEMPO ΩHCHO data and Pandora direct-sun measurements on an annual site average showed good agreement, with an *R*^2^ value of 0.63 across sites for direct-sun and 0.85 for sky-scan. For an additional set of 20 sites with only partial data coverage over the analysis period, the correlation for direct-sun and sky-scan is similar (0.84 and 0.83). Across all differences between Pandora and TEMPO at the 36 sites with annual coverage, 96% fall within TEMPO’s target precision (for 12 co-added pixels) of 1 × 10^16^ molecules cm^−2^ (Szykman et al., 2023).

On a site-by-site basis, individual biases were mainly within ±30%, with 29 of the 36 sites exhibiting *R*^2^ ≥ 0.5. TEMPO showed minimal bias (− 2%) at low ΩHCHO (<1.0 × 10^16^ molecules cm^−2^) but a larger underestimation (−22%) at higher levels (>1.5 × 10^16^ molecules cm^−2^), similar to previous satellite evaluations (e.g., TROPOMI and OMI ΩHCHO). The RMSE increases from ~0.3 to 0.7 × 10^16^ molecules cm^−2^ with ΩHCHO, however the ratio of RMSE to ΩHCHO is twofold lower at higher ΩHCHO, indicating better relative TEMPO performance despite an increasing low bias as column values increase. Pandora agreement with TEMPO strengthens as the relationship improves between direct-sun and sky-scan modes, confirming the need for thorough evaluation of Pandora sites before use in satellite assessments or other scientific applications.

TEMPO effectively captures the seasonal variations in ΩHCHO observed by Pandora with similar correlation between Pandora and TEMPO in each season and a consistent underestimate of higher values. During summer, most sites show maximum ΩHCHO values between 0.6 and 2.3 × 10^16^ molecules cm^−2^ for both TEMPO and Pandora, with winter values being lower by approximately −62% and −66%, respectively. The difference between TEMPO and Pandora remains consistent across seasons for approximately half of the sites, with TEMPO persistently overestimating at some and underestimating at others. TEMPO and Pandora observations show similar diurnal variability in ΩHCHO across most sites with no consistent evidence for a systematic bias in TEMPO in any part of the day or for any diurnal shape. TEMPO and Pandora show consistent behavior across a range of temperature, windspeed, and AOD but with a bias of −26% at AOD > 0.9 likely due to aerosol shielding.

The present work, utilizing Pandora measurements, directly supports bringing the TEMPO ΩHCHO retrieval to full maturity (Szykman et al., 2023). These findings validate the accuracy of TEMPO’s ΩHCHO measurements for enhancing our understanding of photochemical processes and air quality studies. Compared to observations only available at 1p.m., TEMPO increases the number of days with at least one observation by almost 50%. This increase in data availability combined with TEMPO’s excellent performance against Pandora and compared to previous satellite retrievals shows great promise for future scientific applications.

## Supplementary Material

Supplement1

Supporting Information may be found in the online version of this article.

## Figures and Tables

**Table 1:** Diurnal Variation of ΩHCHO Associated With Figure 8

Border color	Diurnal profile shape
Red	Increasing ΩHCHO from morning, peaking in the afternoon, and declining in the evening
Yellow	Increasing ΩHCHO throughout the day
Magenta	Little to no change in ΩHCHO throughout the day
Cyan	Discrepancy between Pandora and TEMPO ΩHCHO

## Data Availability

All data are publicly available. The Pandora data is available at PGN website (https://www.pandonia-global-network.org). Pandora data was accessed through the EPA’s RSIG tool (https://www.epa.gov/hesc/remote-sensing-information-gateway; last access April 2025) (Henderson, 2025). The TEMPO Level 2, version 3 data is available at NASA Earthdata ASDC website (https://search.earthdata.nasa.gov/) and maintained by NASA’s Atmospheric Science Data Center (NASA/LARC/SD/ASDC, 2024). The meteorological data is available at NOAA ISD Lite database (https://www.ncei.noaa.gov/products/land-based-station/integrated-surface-database). The AERONET AOD data is available via NASA GFSC website (https://aeronet.gsfc.nasa.gov/).

## Key Points:

- TEMPO ΩHCHO is well correlated with Pandora observations with an annual average *R*^2^ between 0.63 and 0.85 across 36 Pandora sites
- TEMPO ΩHCHO accurately captures Pandora observed seasonal and diurnal patterns
- Pandora sites must be carefully evaluated using both direct-sun and sky-scan modes to ensure a robust assessment
