# Supplementary material for: Spatiotemporal Assessment of the TEMPO Formaldehyde Column Retrieval Using the Pandonia Global Network
Source: J Geophys Res Atmos. Author manuscript; Available in PMC 2026 May 5. (PMC13137460; doi:10.1029/2025JD044788)
Supplement: Supplement1 [file NIHMS2147927-supplement-Supplement1.pdf]

Supplementary Of

Spatiotemporal Assessment of the TEMPO Formaldehyde Column Retrieval using the Pandonia Global Network

Prajjwal Rawat[1], Katherine R. Travis[1], Barron Henderson[2], James H. Crawford[1], Laura M. Judd[1], Mary Angelique G. Demetillo[1], Tabitha C. Lee[1], David E. Flittner[1], James J. Szykman[2], Lukas C. Valin[2], Andrew Whitehill[2], Eric Baumann[2], Thomas F. Hanisco[3], Apoorva Pandey[3], Gonzalo Gonzalez Abad[4], Caroline R. Nowlan[4], Xiong Liu[4], Kelly Chance[4]

[1]NASA Langley Research Center, Hampton, VA, 23681, USA
[2]Environmental Protection Agency, NC, 27709, USA
[3]NASA Goddard Space Flight Center, Greenbelt, MD 20771, USA
[4]Center for Astrophysics | Harvard & Smithsonian, Cambridge, Massachusetts 02138, USA

Correspondence to: Prajjwal Rawat (prajjwal.rawat@nasa.gov) and Katherine R. Travis (katherine.travis@nasa.gov)

Supplementary Tables

Table S1: Pandora sites in the TEMPO FOR and selection criteria using direct-sun (DS) and sky-scan (SS) mode observation of ΩHCHO observations. In any future studies, it is recommended to reapply these metrics as site selections could change.

No[1]	PGN site	R^2 DS vs SS	MB (%) DS vs SS	Used for TEMPO analysis?	Location (Deg) and Altitute (m)	Local PIs
**Selected PGN sites**					**(Lat, Lon, Alt)**	
1	Pandora52s1_RichmondCA	0.59	21	Yes	(37.91°, -122.35°, 5)	Nader Abuhassan
2	Pandora34s1_MountainViewCA	0.63	31	Yes	(37.42°, -122.06°, 50)	Nader Abuhassan
3	Pandora248s1_TurlockCA	0.85	17	Yes	(37.52°, -120.85°, 45)	Wing To
4	Pandora247s1_WhittierCA	0.71	11	Yes	(33.98°, -118.03°, 130)	Peter Peterson
5	Pandora72s1_SaltLakeCityUT	0.61	27	Yes	(40.73°, -111.87°, 72)	Lukas Valin
6	Pandora204s1_BoulderCO	0.68	49	Yes	(40.04°, -105.24°, 1616)	Tom Hanisco
7	Pandora142s1_MexicoCity-U	0.47	18	Yes	(19.33°, -99.18°, 2280)	Michel Grutter
8	Pandora157s1_MexicoCity-V	0.48	01	Yes	(19.48°, -99.15°, 2255)	Michel Grutter
9	Pandora207s1_ArlingtonTX_	0.70	36	Yes	(32.73°, -97.11°, 15)	Yang Li
10	Pandora25s1_HoustonTX	0.82	30	Yes	(29.72°, -95.34°, 19)	Jimmy Flynn
11	Pandora61s1_AldineTX	0.77	33	Yes	(29.90°, -95.32°, 8)	Tom Hanisco
12	Pandora260s1_CameronLA	0.78	35	Yes	(29.79°, -93.10°, 3)	Mark Phelps
13	Pandora208s1_Windsor-West	0.64	40	Yes	(42.29°, -83.07°, 180)	Thomas Hanisco

No[1]	PGN site	R^2 DS vs SS	MB (%) DS vs SS	Used for TEMPO analysis?	Location (Deg) and Altitute (m)	Local PIs
14	Pandora249s1_ChicagoIL	0.86	20	Yes	(41.97°, -87.71°, 200)	--------
15	Pandora237s1_AtlantaGA-SD	0.85	24	Yes	(33.69°, -84.29°, 250)	Jennifer Kaiser
16	Pandora173s1_AtlantaGA_GA	0.71	24	Yes	(33.78°, -84.40°, 293)	Jennifer Kaiser
17	Pandora158s1_AtlantaGA-CN	0.68	40	Yes	(33.59°, -84.07°, 215)	Jennifer Kaiser
18	Pandora39s1_DearbornMI	0.65	18	Yes	(42.31°, -83.15°, 181)	Lukas Valin
19	Pandora187s1_PittsburghPA	0.73	31	Yes	(40.46°, -79.96°, 265)	Luke Valin
20	Pandora169s1_Egbert	0.64	36	Yes	(44.23°, -79.78°, 251)	Vitali Fioletov
21	Pandora170s1_StGeorge	0.58	34	Yes	(43.66°, -79.40°, 176)	Vitali Fioletov
22	Pandora145s1_Toronto-Scar	0.53	08	Yes	(43.78°, -79.19°, 137)	Vitali Fioletov
23	Pandora70s1_ChapelHillNC	0.59	33	Yes	(35.97°, -79.09°, 50)	James Szykman
24	Pandora140s1_WashingtonDC	0.86	47	Yes	(38.92°, -77.01°, 58)	James Szykman
25	Pandora80s1_BeltsvilleMD_	0.80	16	Yes	(39.05°, -76.88°, 73)	-----
26	Pandora32s1_GreenbeltMD	0.49	07	Yes	(38.99°, -76.84°, 90)	Thomas Hanisco
27	Pandora2s1_GreenbeltMD	0.80	15	Yes	(38.99°, -76.84°, 90)	Thomas Hanisco
28	Pandora156s1_HamptonVA-HU	0.83	49	Yes	(37.02°, -76.33°, 19)	-------
29	Pandora166s1_PhiladelphiaPA	0.79	31	Yes	(39.99°, -75.08°, 6)	Lukas Valin
30	Pandora134s1_BristolPA	0.64	22	Yes	(40.11°, -74.88°, 10)	Lukas Valin
31	Pandora38s1_BayonneNJ_	0.83	21	Yes	(40.67°, -74.12°, 3)	Nader Abuhassan
32	Pandora206s1_BuffaloNY	0.66	46	Yes	(43.00°, -78.79°, 190)	-------
33	Pandora55s1_QueensNY	0.82	35	Yes	(40.73°, -73.82°, 25)	James Szykman
34	Pandora51s1_OldFieldNY	0.72	48	Yes	(40.96°, -73.14°, 3)	Nader Abuhassan
35	Pandora186s1_MadisonCT	0.80	24	Yes	(41.26°, -72.55°, 3)	Lukas Valin
36	Pandora183s1_LondonderryNH	0.72	06	Yes	(42.86°, -71.38°, 108)	Lukas Valin
	**PGN sites with partial annual data**					
1	Pandora258s1_CorpusChrist	0.65	49	No	(27.72°, -97.32°, 14)	--------
2	Pandora261s1_HoustonTX	0.78	27	No	(29.58°, -95.20°, 10)	Madhu Gyawali
3	Pandora143s1_LibertyTX	0.75	27	No	(30.10°, -94.76°, 3)	Yuxuan Wang
4	Pandora259s1_TylerTX	0.81	31	No	(32.34°, -95.41°, 164)	James Flynn
5	Pandora167s1_KenoshaWI	0.77	30	No	(42.50°, -87.81°, 181)	Lukas Valin
6	Pandora66s1_HuntsvilleAL	0.78	26	No	(34.72°, -86.65°, 221)	Nader Abuhassan
7	Pandora253s1_TucsonAZ	0.64	26	No	(32.23°, -110.95°, 779)	-------
8	Pandora57s1_BoulderCO_	0.77	35	No	(39.99°, -105.26°, 1660)	Nader Abuhassan
9	Pandora147s1_SWDetroitMI	0.75	40	No	(42.30°, 83.11°, 178)	Lukas Valin
10	Pandora243s1_Downsview	0.77	45	No	(43.78°, -79.47°, 187)	-------
11	Pandora243s1_Toronto-CNT	0.75	41	No	(43.64°, -79.38°, 330)	--------
12	Pandora31s1_CharlesCityVA	0.74	26	No	(37.32°, -77.20°, 6)	Vickie Connors
13	Pandora255s1_VirginiaBeachVA	0.86	30	No	(37.04°, -76.08°, 20)	John Anderson
14	Pandora69s1_NewBrunswickNJ	0.78	29	No	(40.46°, -74.43°, 19)	Nader Abuhassan
15	Pandora180s1_BronxNY	0.59	29	No	(40.87°, -73.88°, 31)	Luke Valin
16	Pandora236s1_NewLondonCT_	0.56	19	No	(41.37°, -72.10°, 30)	Brooke Stutzman
17	Pandora185s1_EastProvidenceRI	0.67	31	No	(41.84°, -71.36°, 15)	Lukas Valin
18	Pandora155s1_BostonMA_	0.80	12	No	(42.35°, -71.10°, 40)	Jeff Geddes
19	Pandora153s1_ChelseaMA	0.71	49	No	(42.39°, -71.03°, 30)	Jeff Geddes
20	Pandora107s1_LynnMA	0.71	26	No	(42.47°, -70.97°, 52)	---------
	**Low confidence PGN sites**					
1	Pandora74s1_EdwardsCA[2]	NA	NA	No	(34.97°, -117.88°, 692)	James Podolske
2	Pandora68s1_WrightwoodCA_	0.29	45	No	(34.38°, -117.68°, 2207)	Nader Abuhassan

No[1]	PGN site	R^2 DS vs SS	MB (%) DS vs SS	Used for TEMPO analysis?	Location (Deg) and Altitute (m)	Local PIs
3	Pandora181s1_SanJoseCA	0.41	09	No	(37.33°, -121.88°, 69)	Sen Chiao
4	Pandora139s1_SouthJordanUT	0.24	11	No	(40.54°, -112.07°, 1582)	Jeff Geddes
5	Pandora154s1_SaltLakeCityUT	0.32	24	No	(40.76°, -111.84°, 1455)	Lukas Valin
6	Pandora200s1_GrandForksND	0.72	55	No	(47.92°, -97.08°, 256)	Mafany Mongoh
7	Pandora63s1_LaPorteTX	0.41	16	No	(29.67°, -95.06°, 22)	Tom Hanisco
8	Pandora108s1_Toronto-West	0.34	48	No	(43.71°, -79.54°, 141)	Vitali Fioletov
9	Pandora103s1_Downsview	0.28	52	No	(43.78°, -79.47°, 187)	Vitali Fioletov
10	Pandora104s1_Downsview	0.28	164	No	(43.78°, -79.47°, 187)	Vitali Fioletov
11	Pandora135s1_ManhattanNY	0.40	01	No	(40.81°, -73.95°, 34)	Maria Tzortziou
12	Pandora177s1_WestportCT	0.63	55	No	(41.12°, -73.34°, 4)	Lukas Valin
13	Pandora179s1_CornwallCT	0.56	56	No	(41.82°, -73.30°, 505)	Lukas Valin
14	Pandora64s1_NewHavenCT	0.77	51	No	(41.30°, -72.90°, 4)	Nader Abuhassan
15	Pandora26s1_CambridgeMA	0.37	28	No	(42.38°, -71.11°, 60)	Nader Abuhassan
16	Pandora184s1_CapeElizabethME[2]	NA	NA	No	(43.56°, -70.20°, 24)	Lukas Valin

[1]Sites are arranged in order from west to east.

[2]Pandora sites at Edwards, CA and Cape Elizabeth, ME have no matchups (almost all PGN standard data quality flags in 20's). Some PGN sites do not include information about local PIs in the data files.

Table S2. Comparison of Pandora DS and TEMPO ΩHCHO at each selected site, showing mean values with standard deviations, bias, RMSE, and correlation (r^2). Column concentrations, bias and RMSE are in unit of 10^{16} molec cm^{-2}. The corresponding average total column NO_2 from direct sun measurments are also give in the last column. The sites are arranged in the same order as shown in Fig 5.

No[1]	PGN site	Pandora ΩHCHO ± STD (10^{16} molec cm^{-2})	TEMPO ΩHCHO ± STD (10^{16} molec cm^{-2})	Bias (10^{16} molec cm^{-2})	RMSE (10^{16} molec cm^{-2})	R^2	Pandora Total column NO_2 (10^{16} molec cm^{-2})
1	RichmondCA	0.51±0.33	0.59±0.28	0.08	2.99	0.31	0.56
2	LondonderryNH	0.56±0.43	0.66±0.43	0.10	3.15	0.57	0.44
3	Toronto-Scar	0.64±0.48	0.74±0.36	0.09	3.54	0.49	0.89
4	ChapelHillNC	0.72±0.64	0.78±0.48	0.05	5.23	0.36	0.46
5	StGeorge	0.74±0.41	0.62±0.37	-0.12	2.66	0.67	0.94
6	MountainViewCA	0.74±0.32	0.67±0.32	-0.07	2.83	0.40	0.57
7	Egbert	0.78±0.44	0.62±0.34	-0.16	3.41	0.53	0.44
8	MadisonCT	0.78±0.49	0.74±0.48	-0.04	2.69	0.72	0.53
9	SaltLakeCityUT	0.79±0.47	0.58±0.3	-0.20	4.08	0.44	0.80
10	DearbornMI	0.79±0.49	0.80±0.4	0.01	3.02	0.61	1.11
11	GreenbeltMD32	0.83±0.59	1.18±0.69	0.35	6.13	0.49	0.70
12	ChicagoIL	0.84±0.49	0.75±0.4	-0.10	2.76	0.72	1.02
13	BristolPA	0.85±0.54	0.91±0.57	0.060	3.89	0.58	0.81
14	BoulderCO	0.88±0.36	0.49±0.28	-0.38	4.64	0.49	0.57
15	WhittierCA	0.91±0.38	1.21±0.54	0.30	5.13	0.42	1.19
16	BuffaloNY	0.91±0.41	0.71±0.38	-0.19	3.25	0.61	0.54
17	PittsburghPA	0.92±0.5	0.87±0.49	-0.05	3.28	0.62	0.75
18	TurlockCA	0.96±0.41	0.78±0.33	-0.19	3.31	0.56	0.60
19	CameronLA	0.98±0.51	0.86±0.48	-0.13	2.81	0.76	0.36
20	PhiladelphiaPA	1.0±0.68	0.96±0.55	-0.05	3.59	0.73	1.01
21	Windsor-West	1.0±0.4	0.75±0.35	-0.25	3.43	0.68	0.91
22	BeltsvilleMD	1.04±0.73	1.04±0.68	-0.01	4.01	0.71	0.75
23	BayonneNJ	1.06±0.67	0.98±0.56	-0.08	3.78	0.69	1.80
24	GreenbeltMD2	1.11±0.78	1.07±0.7	-0.04	3.83	0.76	0.72
25	QueensNY	1.19±0.7	0.97±0.58	-0.22	4.51	0.68	1.53
26	AtlantaGA-GA	1.24±0.77	1.23±0.79	-0.01	4.95	0.64	0.77
27	OldFieldNY	1.25±0.62	0.83±0.54	-0.43	5.47	0.69	0.70
28	AtlantaGA-CN	1.26±0.66	1.03±0.64	-0.23	4.1	0.75	0.53
29	AtlantaGA-SD	1.26±0.87	1.25±0.82	-0.01	3.93	0.80	0.88
30	ArlingtonTX	1.28±0.65	1.11±0.54	-0.17	4.24	0.64	0.81
31	HamptonVA-HU	1.32±0.65	0.93±0.57	-0.4	5.02	0.77	0.62
32	MexicoCity-V	1.36±0.79	1.14±0.36	-0.21	6.67	0.39	3.10
33	HoustonTX	1.54±0.84	1.28±0.67	-0.25	5.17	0.72	1.15
34	WashingtonDC	1.59±0.82	1.1±0.72	-0.48	6.26	0.77	0.92
35	MexicoCity-U	1.72±0.82	1.35±0.47	-0.36	7.51	0.35	2.30
36	AldineTX	1.74±0.79	1.39±0.71	-0.35	5.99	0.63	0.96

Supplementary Figures

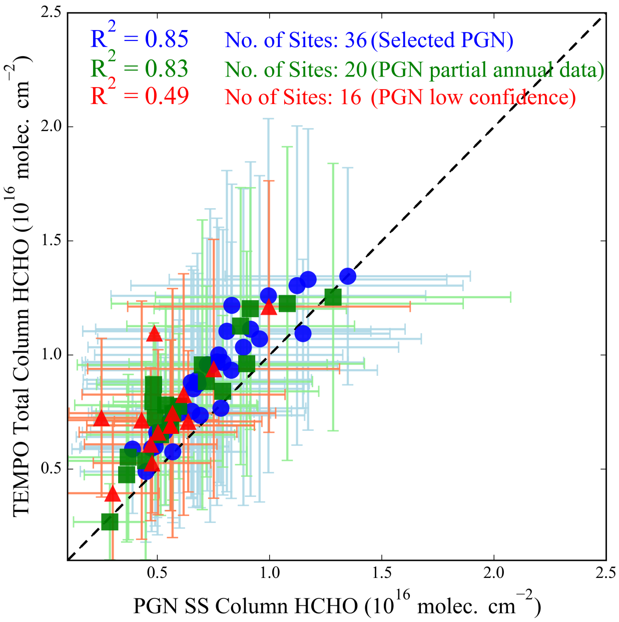

Figure S1. Spatial correlation of TEMPO and Pandora sky scan ΩHCHO during August 2023 to September 2024 at the 36 selected Pandora sites, 20 sites with partial annual data, and 16 low confidence PGN sites.

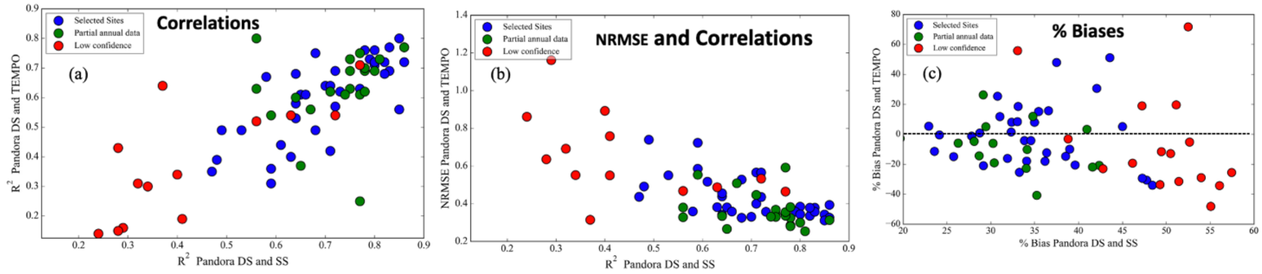

Figure S2. Statistics between Pandora and TEMPO ΩHCHO at all 72 Pandora locations for (a) correlation relationship between R^2 of Pandora direct-sun and sky-scan and R^2 between Pandora direct-sun and TEMPO (b) normalized RMSE between TEMPO and Pandora direct-sun versus the R^2 between Pandora direct-sun and sky-scan and (c) the % bias between Pandora DS and TEMPO versus the %bias between Pandora DS and SS.

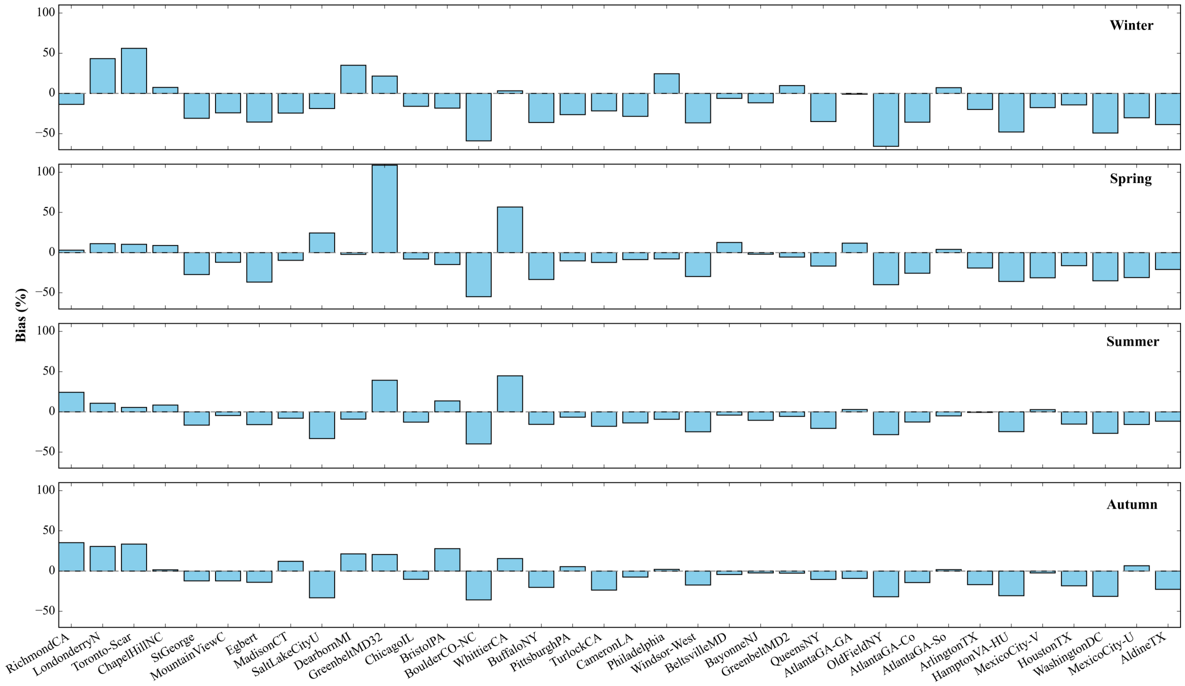

Figure S3. Percentage biases in ΩHCHO between TEMPO v3 and Pandora direct-sun measurements during August 2023 to September 2024 in four seasons. The seasons are described as Winter (DJF), Spring (MAM), Summer (JJA), and Autumn (SON), respectively. The sites are arranged in the same order as shown in Fig 5.

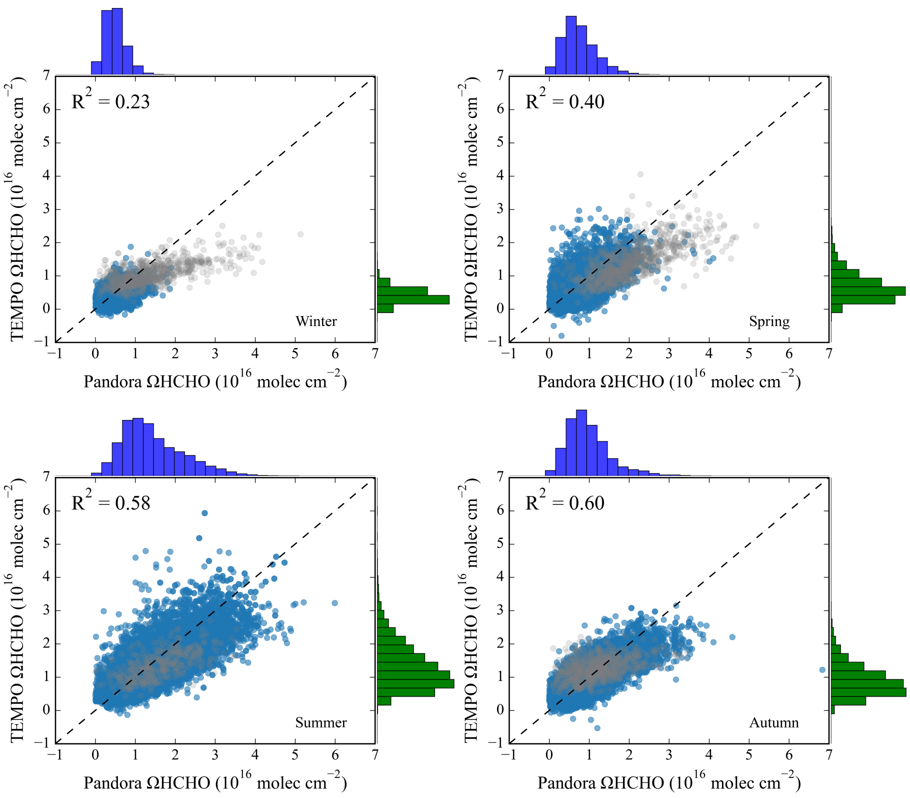

Figure S4. Seasonal scatter plot and density distribution of ΩHCHO between TEMPO v3 and Pandora direct-sun measurements during August 2023 to September 2024 for all hourly collocations. The seasons are described as Winter (DJF), Spring (MAM), Summer (JJA), and Autumn (SON), respectively. TEMPO (green) and Pandora (blue) distributions are shown on adjacent axis. The two Mexico City sites are shown separately with gray points to highlight their distinct seasonal patterns.

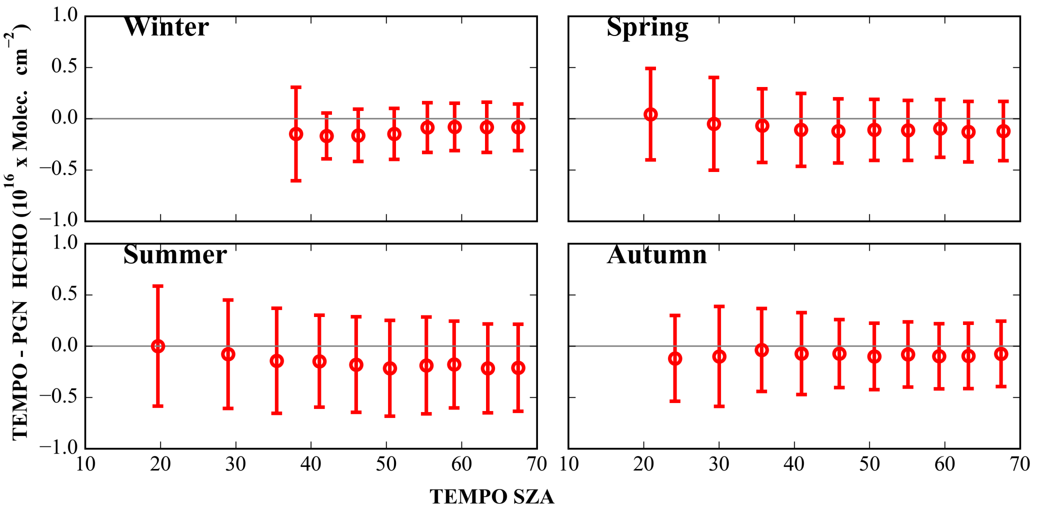

Figure S5. ΩHCHO difference between TEMPO and Pandora direct-sun for different seasons along the varying SZA of TEMPO. The mean and standard deviation is calculated at 10 decile bins of SZA parameter. Both Mexico City sites are excluded due to their weaker seasonal patterns.

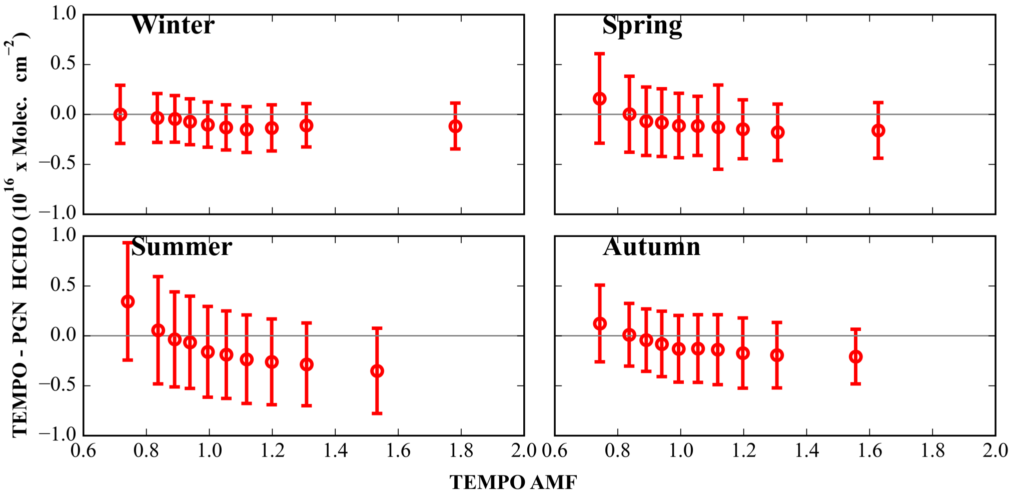

Figure S6. ΩHCHO difference between TEMPO and Pandora direct-sun for different seasons along the varying AMF of TEMPO. The mean and standard deviation is calculated at 10 decile bins of AMF parameter. Both Mexico City sites are excluded due to their weaker seasonal patterns.

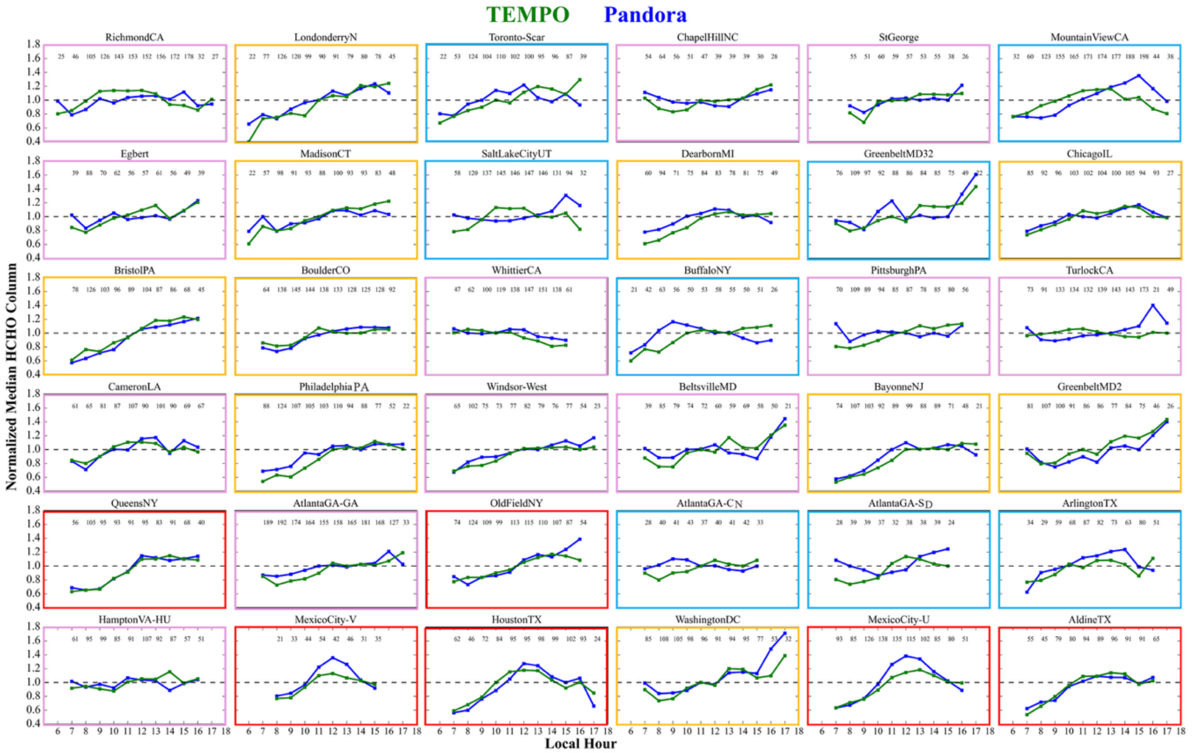

Figure S7. Normalized diurnal variation of ΩHCHO from TEMPO (green) and Pandora sky scan (blue) measurements during ozone season (May to October) for all hourly spatio-temporal co-locations. The number of matchups at each hour is also annotated in the top of each subplot. The subplots are categorized into distinct groups based on their diurnal patterns**,** with red, yellow, magenta, and cyan borders indicating different characteristic behaviors given in Table 1. The sites are arranged in the same order as shown in Figure 5. Pandora SS provides more matchups due to its ability to retrieve data using both open filter and U340 filter modes compared to direct sun (Cede et al., 2025; Rawat et al., 2024). Add about color and but bufflo in cyan.

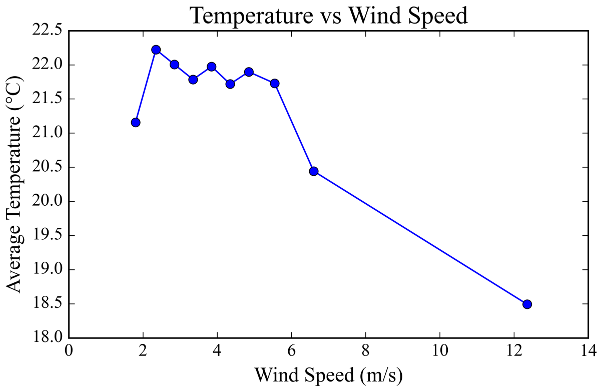

Figure S8. Relationship between average temperature along different percentile bins of wind speed, showing the variation of average temperature with increasing wind speed.
